# The taxonomy, host range and pathogenicity of coronaviruses and other viruses in the *Nidovirales* order

**DOI:** 10.1186/s44149-021-00005-9

**Published:** 2021-04-23

**Authors:** Zhijian Zhou, Ye Qiu, Xingyi Ge

**Affiliations:** Hunan Provincial Key Laboratory of Medical Virology, Institute of Pathogen Biology and Immunology, College of Biology, Hunan University, 27 Tianma Rd., Changsha, Hunan China

**Keywords:** Coronavirus, *Nidovirales*, Hosts, S protein, Genetics, Evolution

## Abstract

The frequent emergence of coronavirus (CoV) epidemics has seriously threatened public health and stock farming. The major hosts for CoVs are birds and mammals. Although most CoVs inhabit their specific natural hosts, some may occasionally cross the host barrier to infect livestock and even people, causing a variety of diseases. Since the beginning of the new century, increasing attention has been given to research on CoVs due to the emergence of highly pathogenic and genetically diverse CoVs that have caused several epidemics, including the recent COVID-19 pandemic. CoVs belong to the *Coronaviridae* family of the *Nidovirales* order. Recently, advanced techniques for viral detection and viral genome analyses have enabled characterization of many new nidoviruses than ever and have greatly expanded the *Nidovirales* order with new classification and nomenclature. Here, we first provide an overview of the latest research progress in the classification of the *Nidovirales* order and then introduce the host range, genetic variation, genomic pattern and pathogenic features of epidemic CoVs and other epidemic viruses. This information will promote understanding of the phylogenetic relationship and infectious transmission of various pathogenic nidoviruses, including epidemic CoVs, which will benefit virological research and viral disease control.

## Introduction

The ongoing coronavirus disease 2019 (COVID-19) pandemic caused by severe acute respiratory syndrome coronavirus 2 (SARS-CoV-2) has been prevalent in almost all regions of the world, resulting in 129,471,273 confirmed cases and 2,825,407 deaths by April 1, 2021. COVID-19 was declared as a worldwide pandemic on February 20, 2020 (WHO n.d.). During the last two decades, 5 types of coronaviruses (CoV) have been found to infect humans, including SARS-CoV (2003) (Falsey and Walsh 2003), human coronavirus NL63 (HCoV-NL63, 2004) (van der Hoek et al. 2004), human coronavirus HKU1 (HCoV-HKU1, 2005) (Woo et al. 2005), Middle East Respiratory Syndrome Coronavirus (MERS-CoV, 2012) (Zaki et al. 2012), and SARS-CoV-2 (2019) (Zhu et al. 2020), among which SARS-CoV, MERS-CoV, and SARS-CoV-2 are highly pathogenic CoVs. Together with 2 human CoVs (HCoV-229E and HCoV-OC43) discovered in the 1960s, 7 types of CoVs have been found to infect humans to date (Vetterlein and Hesse 1965; McIntosh et al. 1967). The frequent global CoV pandemics in the new century have alarmed experts and indicated the great threat of pathogenic CoVs to public health worldwide.

In addition to human CoVs, many animal CoVs also cause diseases in domestic animals and serious harm to livestock farming. For example, porcine CoVs are common porcine pathogens, including transmissible gastroenteritis virus (TGEV) (Doyle and Hutchings 1946), porcine respiratory coronavirus (PRCV, Chen et al. 2019), porcine epidemic diarrhea virus (PEDV, Pensaert and Bouck 1978), porcine deltacoronavirus (PDCoV, Woo et al. 2012), porcine hemagglutinating encephalomyelitis virus (PHEV), and porcine enteric alphacoronavirus (PEAV), also known as swine acute diarrhea syndrome coronavirus (SADS-CoV, Gong et al. 2017; Pan et al. 2017; Zhou et al. 2018). In addition to porcine CoVs, there are CoVs that infect other livestock and animals, such as bovine coronavirus (BCoV, Castells et al. 2017), equine coronavirus (ECoV, Pusterla et al. 2016), canine coronavirus (CCoV, Decaro et al. 2010), and feline coronavirus (FCoV, Li et al. 2019). Furthermore, some avian CoVs, such as infectious bronchitis virus (IBV) and the closely related turkey coronavirus (TCoV) (Jackwood et al. 2012; Brown et al. 2016) cause diseases in poultry.

After the SARS-CoV epidemic in 2003, studies tracing epidemic CoVs revealed the extensive presence of nonpathogenic CoVs with remarkable genetic diversity in many species of wild animals. The natural hosts of these CoVs include bats, rodents, cats, wild birds, marine mammals and so on (Woo et al. 2012; Ge et al. 2013; Ge et al. 2017; Hu et al. 2017). According to genomic sequencing and phylogenetic analyses, most human CoVs have been shown to originate from wild animals at different time points and through different paths (Cui et al. 2019; Zhou et al. 2020b). It’s particularly noteworthy that the 3 highly pathogenic CoVs, including SARS-CoV, MERS-CoV and SARS-CoV-2, may originate from bats (Ge et al. 2013; Azhar et al. 2014; Wang et al. 2014b; Zhou et al. 2020b). The HKU-2-related bat coronavirus, named PEAV/SADS-CoV, was recently transmitted from bats to swine and caused an epidemic in 2016-17 (Zhou et al. 2018). On the other hand, the long-term persistence and rapid mutagenesis of native CoVs may lead to the emergence of new types of epidemic CoVs, which has brought many challenges to the prevention and control of these diseases. For example, epidemiological studies have shown an increasing genotype diversity of PEDV in pigs and IBV in poultry (Zhou et al. 2014; Li et al. 2017; Zeng et al. 2017). At the same time, the diversity and evolution of CoVs in their natural reservoirs, such as bats and birds, have also caused increasing concerns about their potential for interspecific transmission and to cause epidemics (Hu et al. 2017).

CoVs are membrane-enveloped viruses with a linear, single-stranded and positive RNA genome. CoVs belong to the *Orthocoronavirinae* subfamily of the *Coronaviridae* family in the *Cornidovirineae* suborder of the *Nidovirales* order (https://talk.ictvonline.org/taxonomy/) (Walker et al. 2019). After the SARS epidemic in 2003, a large number of CoVs with high genetic diversity were identified in various natural hosts of birds and mammals that could not be classified using the former taxonomic system. Thus, the system for CoV classification has been adjusted several times, and the latest version was proposed (International Committee on Taxonomy of Viruses Executive 2020). In addition, the widespread nature of CoVs raises concerns about the spread, spillover and prevalence of epidemic viruses in the future, which may impose significant respiratory and/or gastrointestinal diseases on humans and economic losses to livestock farming. Therefore, it is critical to investigate the characteristics of transmission and infection of CoVs. In this review, we provide an overview of the latest classification of viruses in the *Nidovirales* order and then focus on the genetic evolution, pathogenesis, and cross-species transmission of epidemic CoVs and other epidemic nidoviruses, aiming to provide a comprehensive understanding of the current progress of studies concerning CoVs and nidoviruses.

To avoid any divergence, information about the viruses listed in this review is preferentially in accordance with the latest update in the taxonomy system of the International Committee on Taxonomy of Viruses Executive on Taxonomy of Viruses (ICTV) (https://talk.ictvonline.org/taxonomy/), if available, which may not be completely consistent with the description of the original literature. At the same time, only the classified virus strains listed on the ICTV website are involved, while some virus strains reported elsewhere may not be mentioned. In addition, at present, the origin and spread of nidoviruses are still controversial topics, and some descriptions in this review may represent the authors’ personal views only.

## Summary of the *Nidovirales* order

### Taxonomy of the *Nidovirales* order

**Fig. 1:**
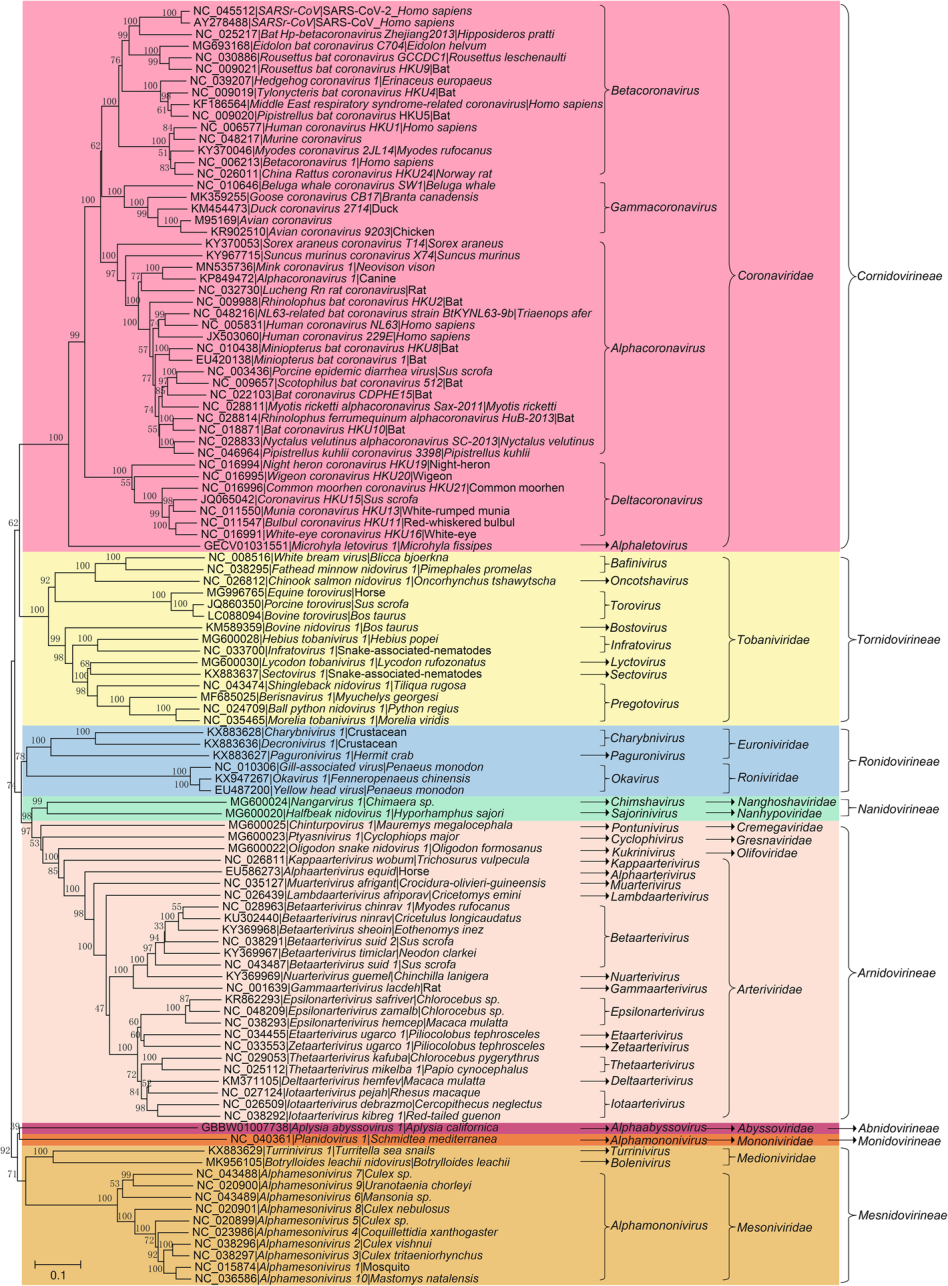
Phylogenetic tree of RNA-dependent RNA polymerase (RdRp) amino acid sequences of 109 viral species in the *Nidovirales* order. The tree was constructed using the neighbor-joining method with a p-distance model and 1000 bootstraps in the MEGA V. 7.0.14

The *Nidovirales* order was first proposed by ICTV in 1996 and was named after the Latin term *nido,* which means “nest” (Pringle 1996). Initially, the order contained only 2 viral families, *Coronaviridae* and *Arteriviridae* (Pringle 1996). However, with improvements in techniques of virus detection and viral metagenomics, additional viral genomes have been detected (Ge et al. 2012; Shi et al. 2018). Characterization of these novel viral genomes has profoundly changed the classification of viruses. At present, 8 suborders have been established under the *Nidovirales* order: *Abnidovirineae*, *Arnidovirineae*, *Cornidovirineae*, *Mesnidovirineae*, *Monidovirineae*, *Nanidovirineae*, *Ronidovirineae,* and *Tornidovirineae* (Walker et al. 2019). These 8 suborders contain 14 viral families, 25 subfamilies, 39 genera, 65 subgenera, and a total of 109 viral species (Tables 1, 2 and 3). Genetic relationship among the 109 representative virus species is shown in Fig. 1.

**Table 1:** Hosts and sampled countries for *Cornidovirineae* viruses

Subfamily	Genus	Subgenus	Host	Country
***Letovirinae***	*Alphaletovirus*	*Milecovirus*	*Microhyla fissipes*	Unknown
***Orthocoronavirinae***	*Alpha-coronavirus*	*Colacovirus*	Bat	USA, Canada
		*Decacovirus*	Bat	China
		*Duvinacovirus*	*Homo sapiens*, Camel, Bat	USA, Netherlands, Italy, Sweden, Ghana, Saudi Arabia, Germany, Kenya, Haiti, United Arab Emirates
		*Luchacovirus*	Rat	China, United Kingdom
		*Minacovirus*	Mink, *Neovison vison*	USA, Netherlands, Japan, China, Denmark
		*Minunacovirus*	Bat	China
		*Myotacovirus*	*Myotis ricketti*	China
		*Nyctacovirus*	Bat	China, Italy
		*Pedacovirus*	*Sus scrofa*	China, South Korea, USA, Mexico, Viet Nam, Canada, Ukraine, Belgium, France, Italy, Thailand, Viet Nam, Slovenia, Colombia, United Kingdom, Hungary, Japan, Germany, Russia, Philippines, Spain
		*Rhinacovirus*	Bat, *Sus scrofa*	China
		*Setracovirus*	*Homo sapiens*	Netherlands, USA, China, Haiti, Kenya, South Korea
		*Soracovirus*	*Sorex araneus*	China
		*Sunacovirus*	*Suncus murinus*	China
		*Tegacovirus*	*Sus scrofa*, Dog, Cat	USA, China, Italy, Netherlands, Germany, United Kingdom, Belgium, Denmark, Mexico, Brazil, Spain
	*Beta-coronavirus*	*Embecovirus*	*Homo sapiens*, Bovine, Alpaca, Antelope, Deer, Rat, *Oryctolagus cuniculus*, Dog, Camel, *Sus scrofa*, Horse, *Pan troglodytes verus*	Japan, Australia, USA, France, Belgium, China, Germany, South Korea, United Arab Emirates, Saudi Arabia, United Kingdom, Bangladesh, Mexico, Malaysia, Cote d'Ivoire, Uganda, Thailand, Kenya, Ethiopia, Morocco, Nigeria
		*Hibecovirus*	Bat	Nigeria, China
		*Merbecovirus*	Bat, *Homo sapiens*, Camel, Hedgehog, *Lama glama*	China, United Kingdom, Germany, Jordan, South Africa, Saudi Arabia, United Arab Emirates, France, Qatar, Egypt, USA, South Korea, Oman, Thailand, Italy, Ethiopia, Morocco, Burkina Faso, Nigeria, Kenya
		*Nobecovirus*	Bat	China, India, Singapore, Cameroon
		*Sarbecovirus*	Bat, Civet, *Homo sapiens*, *Malayan pangolin*, *Felis catus,* Mink, Dog, Tiger	Global
	*Delta-coronavirus*	*Andecovirus*	Wigeon	China
		*Buldecovirus*	*Sus scrofa*, Birds, *Leopard cat*	USA, South Korea, China, Thailand, Laos, Viet Nam, Japan, United Arab Emirates, Poland, Australia
		*Herdecovirus*	Night-heron	China
	*Gamma-coronavirus*	*Brangacovirus*	*Branta canadensis*	Canada
		*Cegacovirus*	*Delphinapterus leucas*, *Bottlenose dolphin*	USA
		*Igacovirus*	Chicken, Turkey, Duck, Pheasant, Goose, Partridge, Pigeon, Peafowl	China, USA, Canada, Netherlands, Nigeria, Sweden, South Korea, Australia, Ukraine, South Africa, Italy, Belgium, France, India, Poland, Brazil, Sudan, Jordan, Pakistan, Egypt, United Kingdom, Uruguay, Iran, Malaysia, Peru, Australia, Norway

Notes: Some related but unclassified viruses are not listed

**Table 2:** Hosts and sampled countries of *Arnidovirineae* viruses

Family	Subfamily	Genus	Subgenus	Host	Country
***Arteriviridae***	*Crocarterivirinae*	*Muarterivirus*	*Muarterivirus afrigant*	*Crocidura olivieri guineensis*	Guinea
	*Equarterivirinae*	*Alphaarterivirus*	*Alphaarterivirus equid*	Horse, Donkey	USA, France, United Kingdom, Chile, Serbia, Poland
	*Heroarterivirinae*	*Lambdaarterivirus*	*Lambdaarterivirus afriporav*	*Cricetomys emini*	Cameroon
	*Simarterivirinae*	*Deltaarterivirus*	*Hedartevirus*	Monkey	USA
		*Epsilonarterivirus*	*Sheartevirus*	Monkey	USSR, South Africa
		*Etaarterivirus*	*Etaarterivirus ugarco 1*	Monkey	Uganda
		*Iotaarterivirus*	*Debiartevirus*	Monkey	Cameroon
			*Kigiartevirus*	Monkey	Uganda
			*Pedartevirus*	Monkey	USA
		*Thetaarterivirus*	*Kaftartevirus*	Monkey, Papio	Zambia
			*Mitartevirus*	Papio	Tanzania
		*Zetaarterivirus*	*Zeta-arterivirus ugarco 1*	Monkey	Uganda
	*Variarterivirinae*	*Betaarterivirus*	*Ampobartevirus*	*Sus scrofa*	USA, South Korea, India, China
			*Chibartevirus*	*Myodes rufocanus, Eothenomys inez*	China
			*Eurpobartevirus*	*Sus scrofa*	Netherlands, France, Hungary, China, Russia
			*Micartevirus*	*Neodon clarkei*	China
		*Gammaarterivirus*	*Gammaarterivirus lacdeh*	Rat	Unknown
		*Nuarterivirus*	*Nuarterivirus guemel*	*Chinchilla lanigera*	China
	*Zealarterivirinae*	*Kappaarterivirus*	*Kappaarterivirus wobum*	*Trichosurus vulpecula*	Australia, New Zealand
***Cremegaviridae***	*Rodepovirinae*	*Pontunivirus*	*Chinturpovirus 1*	*Mauremys megalocephala*	China
***Gresnaviridae***	*Reternivirinae*	*Cyclophivirus*	*Ptyasnivirus 1*	*Cyclophiops major*	China
***Olifoviridae***	*Gofosavirinae*	*Kukrinivirus*	*Oligodon snake nidovirus 1*	*Oligodon formosanus*	China

Notes: Some related but unclassified viruses are not listed

**Table 3:** Hosts and sampled countries of *Ab-*, *Mes-*, *Mo-*, *Na-*, *Ro-* and *Tor-Nidovirineae* viruses

Suborders	Family	Subfamily	Genus	Subgenus	Host	Country
**Abnidovirineae**	*Abyssoviridae*	*Tiamatvirinae*	*Alphaabyssovirus*	*Aplyccavirus*	*Aplysia californica*	Unknown
***Mesnidovirineae***	*Medioniviridae*	*Medionivirinae*	*Turrinivirus*	*Beturrivirus*	*Turritella sea snails*	China
		*Tunicanivirinae*	*Bolenivirus*	*Balbicanovirus*	*Botrylloides leachii*	New Zealand
	*Mesoniviridae*	*Hexponivirinae*	*Alphamesonivirus*	*Casualivirus*	*Coquillettidia xanthogaster*	Australia
				*Enselivirus*	Culex	Cote d'Ivoire
				*Hanalivirus*	Culex	Cote d'Ivoire
				*Kadilivirus*	Culex	Brazil
				*Karsalivirus*	Culex	Viet Nam, Indonesia
				*Menolivirus*	Uranotaenia	Cote d'Ivoire
				*Namcalivirus*	Aedes, Rat, Culex, Uranotaenia	Viet Nam, Cote d'Ivoire, Indonesia, Thailand, China, South Korea, Senegal, Ghana, Australia, Italy, USA, Austria, Mexico, Sweden, Brazil, Spain
				*Ofalivirus*	Mansonia	Brazil
***Monidovirineae***	*Mononiviridae*	*Mononivirinae*	*Alphamononivirus*	*Dumedivirus*	*Schmidtea mediterranea*	USA
***Nanidovirineae***	*Nanghoshaviridae*	*Chimanivirinae*	*Chimshavirus*	*Nangarvirus 1*	*Chimaera sp.*	China
	*Nanhypoviridae*	*Hyporhamsavirinae*	*Sajorinivirus*	*Halfbeak nidovirus 1*	*Hyporhamphus sajori*	China
***Ronidovirineae***	*Euroniviridae*	*Ceronivirinae*	*Charybnivirus*	*Cradenivirus*	*Charybdis crab*	China
				*Wenilivirus*	Crustacean	China
		*Crustonivirinae*	*Paguronivirus*	*Behecravirus*	*Hermit crab*	China
	*Roniviridae*	*Okanivirinae*	*Okavirus*	*Tipravirus*	Penaeus	Australia, Thailand, Egypt, China
***Tornidovirineae***	*Tobaniviridae*	*Piscanivirinae*	*Bafinivirus*	*Blicbavirus*	*Blicca bjoerkna*	Germany
				*Pimfabavirus*	*Pimephales promelas*	USA
				unclassified *Bafinivirus*	*Salmo salar, Yellow catfish*	Canada, China
			*Oncotshavirus*	*Salnivirus*	*Oncorhynchus tshawytscha, Macrognathus aculeatus, Crucian carp, Carassius auratus*	Canada, China, United Kingdom
		*Remotovirinae*	*Bostovirus*	*Bosnitovirus*	*Cattle*	USA
		*Serpentovirinae*	*Infratovirus*	*Hepoptovirus*	*Hebius popei*	China
				*Xintolivirus*	*Snake-associated nematodes*	China
			*Lyctovirus*	*Rebatovirus*	*Lycodon rufozonatus*	China
			*Pregotovirus*	*Roypretovirus*	*Python regius, Morelia viridis*	USA, Switzerland
				*Snaturtovirus*	*Myuchelys georgesi*	Australia
				*Tilitovirus*	*Tiliqua rugosa*	Australia
			*Sectovirus*	*Sanematovirus*	*Snake-associated nematodes*	China
		*Torovirinae*	*Torovirus*	*Renitovirus*	*Sus scrofa*, Cattle, Horse	China, USA, Japan, Switzerland

Notes: Some related but unclassified viruses are not listed

### Common features of viruses in the *Nidovirales* order

**Fig. 2:**
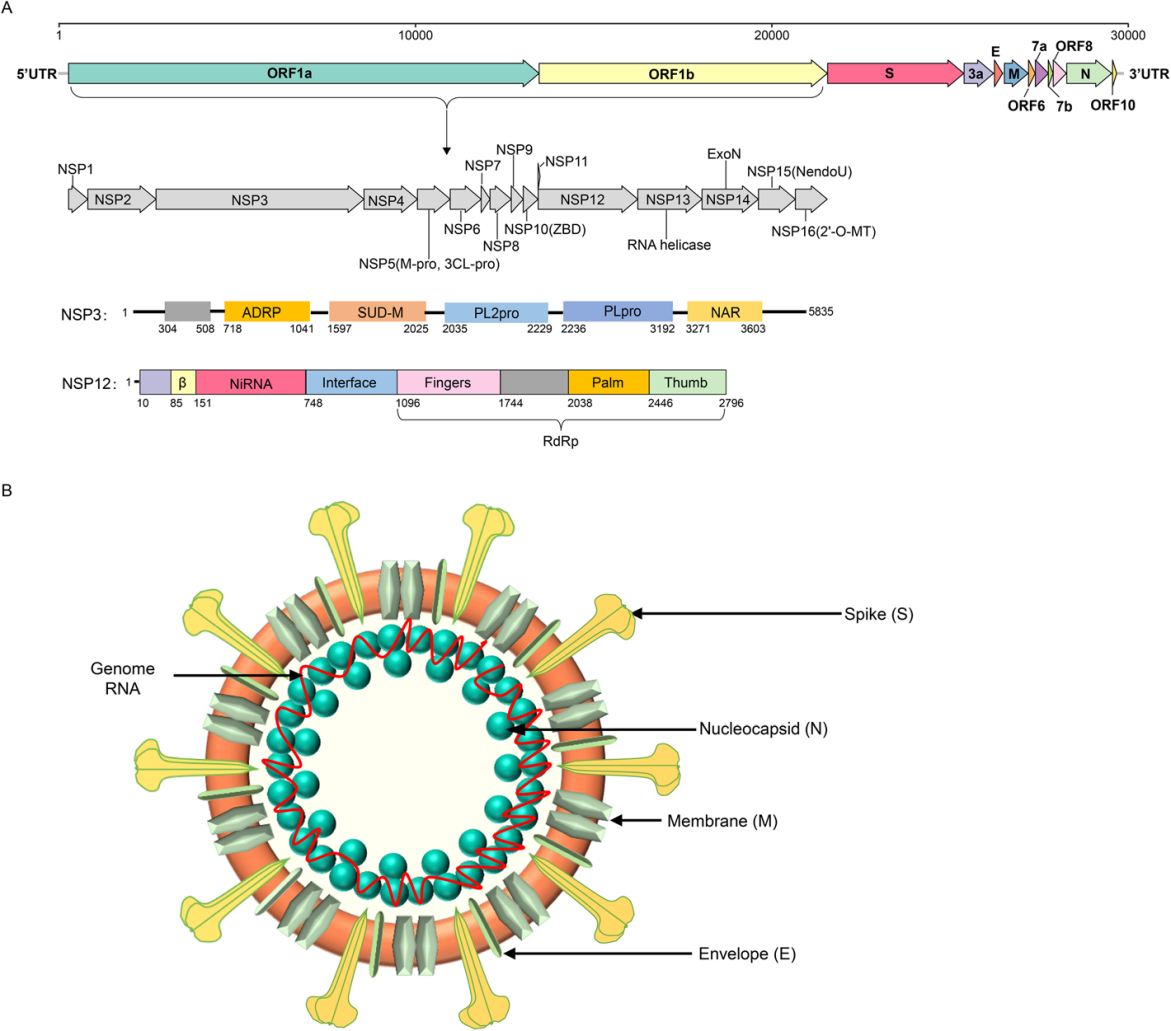
Genome structure, coding proteins, and viral particle structure diagram of coronavirus represented by SARS-CoV-2. **a** Genome structure, functional domains and locations of SARS-CoV-2. NSP, nonstructural proteins; 3Clpro, 3C-like protease; NiRAN, nidovirus RdRp-associated nucleotidyltransferase; RdRp, RNA-directed RNA polymerase; ZBD, Zn-binding domain covalently linked to HEL1; HEL1, helicase of superfamily 1; NendoU, endonuclease; 2′-O-MT, 2′-O-methyltransferase; ExoN, 3′-5′ exonuclease; SUD-M, SARS-unique domains; ADRP, ADP-ribose-1″-phosphatase; PLpro, papain-like protease; NAR, nucleic acid-binding domain. **b** Viral particle diagram of SARS-CoV-2. Structural proteins of S, N, M, and E are labeled, and the red line indicates the RNA genome

Members of the *Nidovirales* order are membrane-enveloped viruses with a single strand positive RNA genome. Although the viral particle size, morphology, and genome size of viruses in these 8 suborders vary greatly, nidoviruses share some significant common features. The most typical feature is transcription of multiple 3'-nested subgenomic RNAs from the 5’ terminus to the 3’ terminus along the genome during viral gene expression, which endows the order name of *nido*-“nest” (Posthuma et al. 2006). The other common features include similar genomic organization (encoding the nonstructural polyprotein upstream and the structural protein downstream of the genome), expression of the polyprotein by ribosomal frame shifting mode, and some nonstructural proteins (NSPs) with unique protease activities (Fig. 2) (Posthuma et al. 2008). Due to the complexity of the genome, the new classification standard of order *Nidovirales* is based on amino acid (aa) sequences of several hallmark genes only, which include 3CLpro (3C-like protease), NiRAN (nidovirus RdRp-associated nucleotidyltransferase), RdRp (RNA-directed RNA polymerase), ZBD (Zn-binding domain covalently linked to HEL1), and HEL1 (helicase of superfamily 1) domains of the replicase protein (International Committee on Taxonomy of Viruses Executive 2020). Domains of 3CLpro, NiRAN, RdRp, ZBD, and HEL1 located in the genome and proteins are illustrated in Fig. 2 using the SARS-CoV-2 model.

## The taxonomy, host range and pathogenicity of *Cornidovirineae*

### Suborder *Cornidovirineae*

**Fig. 3:**
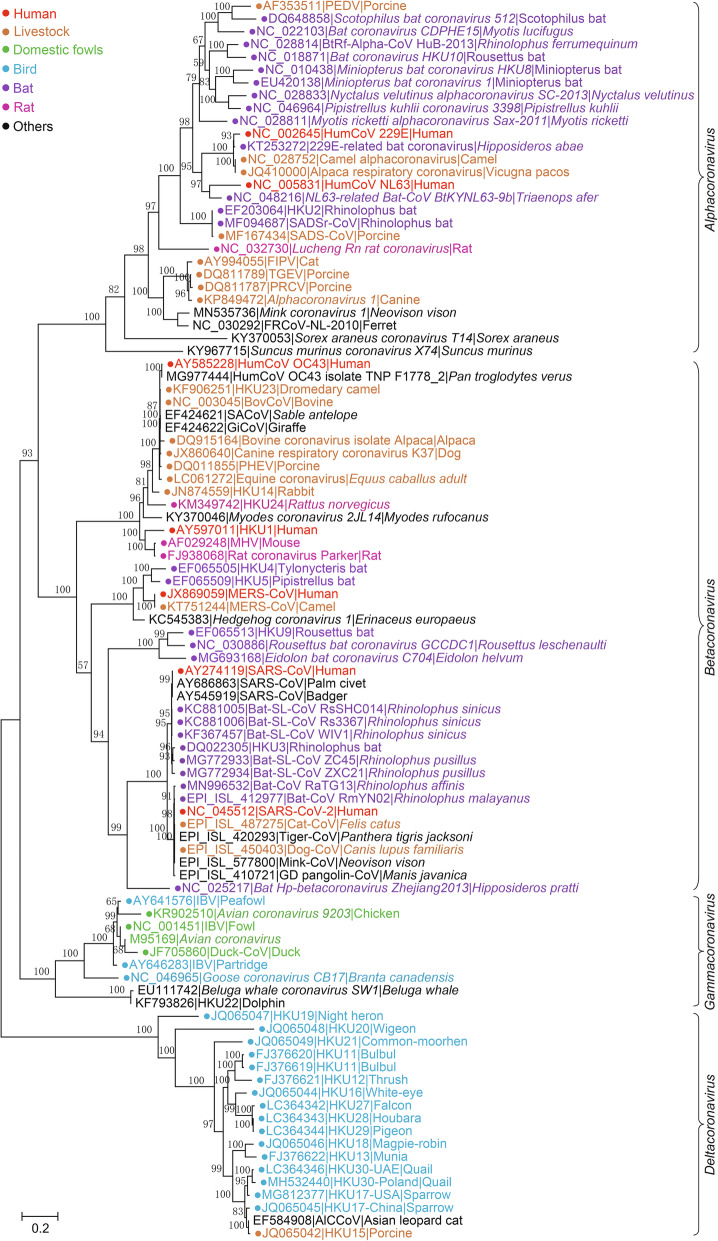
The maximum likelihood tree of coronaviruses based on amino acid sequences of RdRp gene. The tree was constructed using an IQ-tree with 10,000 ultrafast bootstraps and the most appropriate substitution model of LG+F+I+G4, which was calculated by ModelFinder

**Fig. 4:**
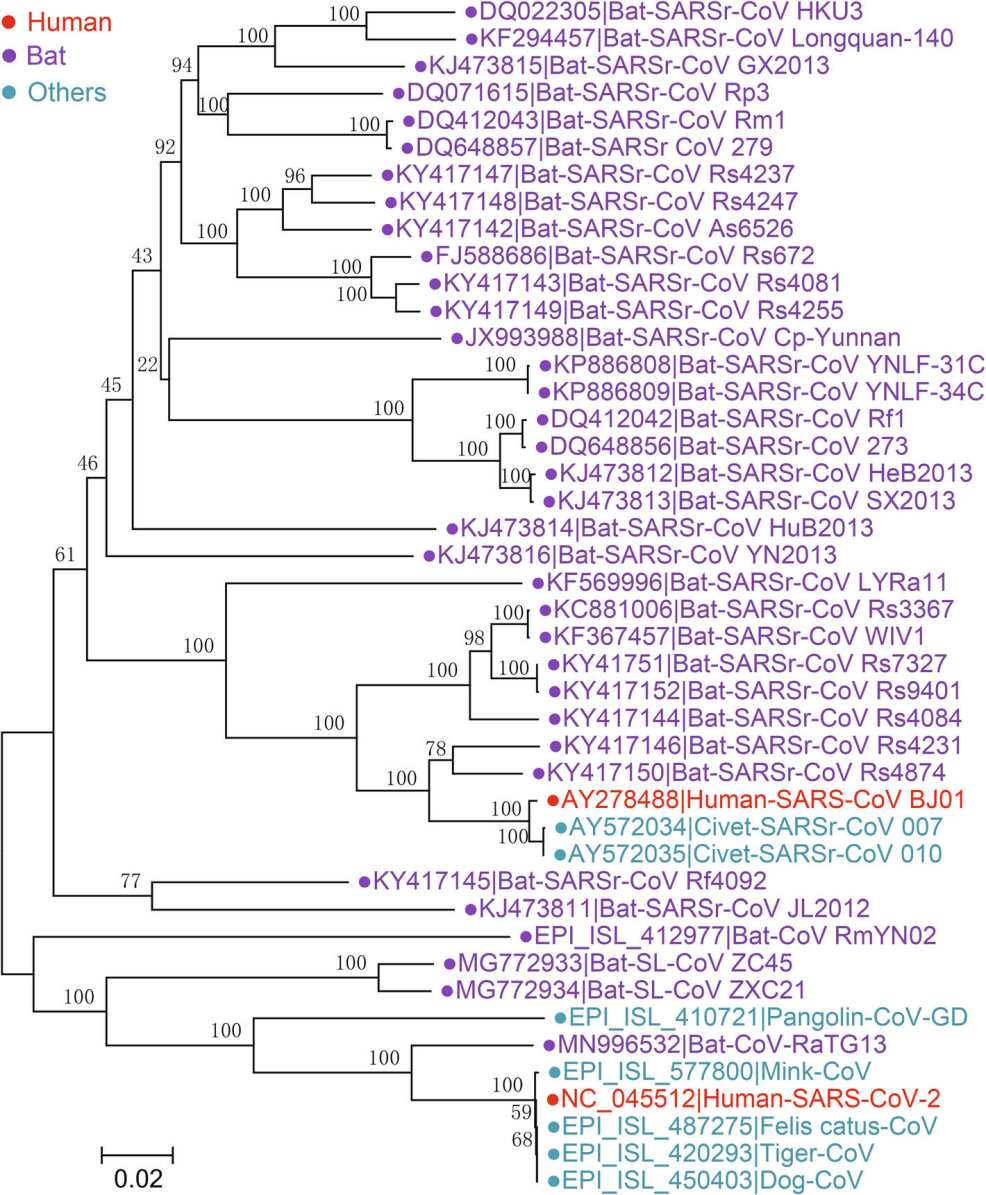
The neighbor-joining tree of spike gene nucleotide sequences in *Sarbecovirus*. The tree was constructed using the p-distance model and 1000 bootstraps in the MEGA V. 7.0.14. Viral strains derived from humans, bats, and other animals are indicated by red, purple, and cyan, respectively

**Fig. 5:**
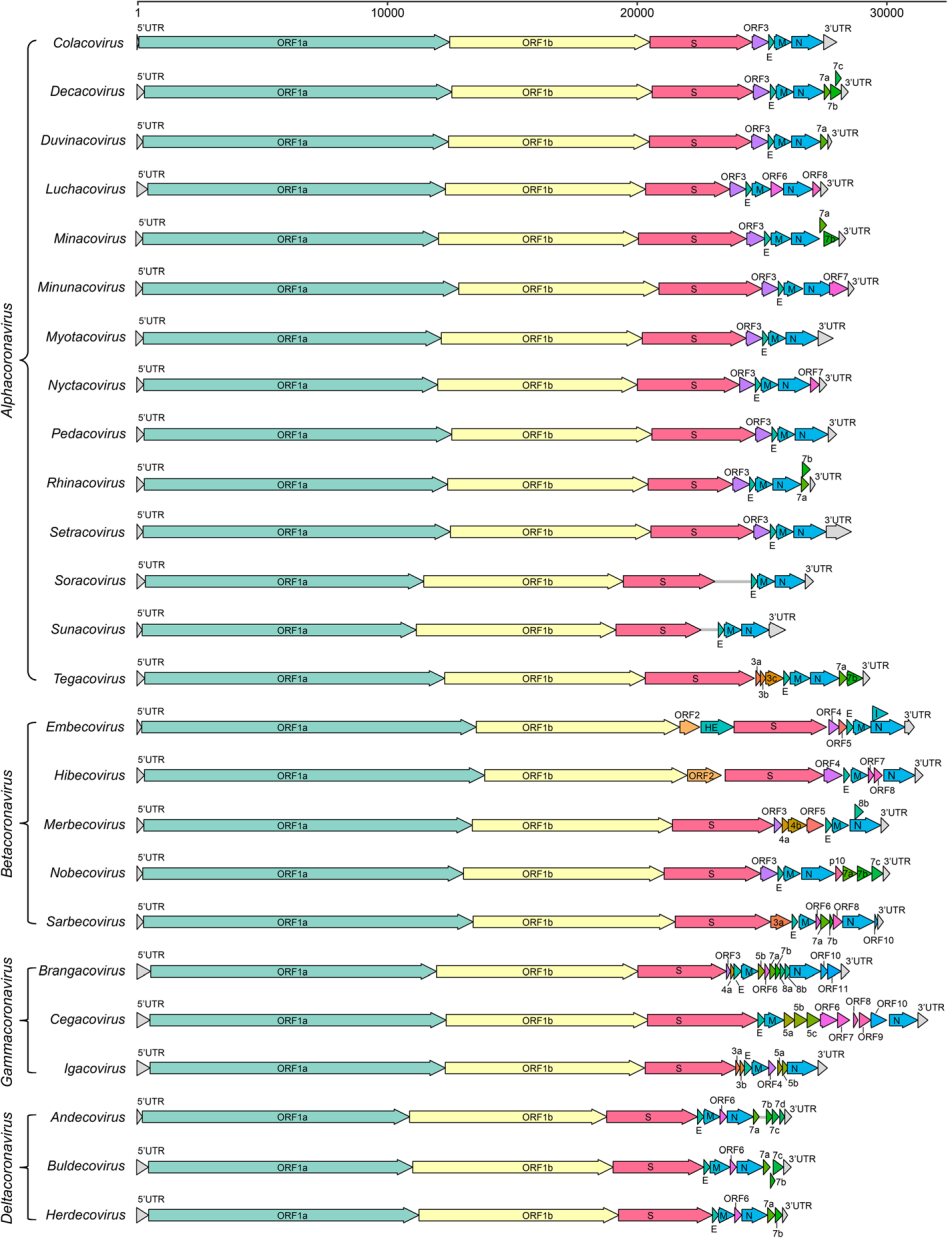
Gene composition of 25 subgenera in 4 viral genera of *Orthocoronavirinae*. The reference strains used for mapping in each subgenus are as follows: *Colacovirus* (Bat-CoV CDPHE15/USA/2006, NC_022103.1), *Decacovirus* (Bat-CoV HKU10, NC_018871.1), *Duvinacovirus* (229E-related bat-CoV BtKY229E-1, KY073747.1), *Luchacovirus* (AcCoV-JC34, NC_034972.1), *Minacovirus* (FRCoV-NL-2010, NC_030292.1), *Minunacovirus* (Bat-CoV HKU8, NC_010438.1), *Myotacovirus* (BtMr-CoV/SAX2011, NC_028811.1), *Nyctacovirus* (Bat-CoV HKU33, MK720944.1), *Pedacovirus* (PEDV, NC_003436.1), *Rhinacovirus* (SADS-CoV, MT199592.1), *Setracovirus* (NL63-related bat-CoV BtKYNL63-9b, NC_048216.1), *Soracovirus* (Shrew-CoV/Tibet2014, KY370053.1), *Sunacovirus* (Wencheng Sm shrew CoV Xingguo-74, KY967715.1), *Tegacovirus* (CCoV/NTU336/F/2008, GQ477367.1), *Embecovirus* (Murine CoV, JX169867.1), *Hibecovirus* (Bat Hp-betacoronavirus/Zhejiang2013, NC_025217.1), *Merbecovirus* (Human MERS-CoV, NC_019843.3), *Nobecovirus* (Rousettus bat-CoV GCCDC1 356, NC_030886.1), *Sarbecovirus* (SARS-CoV-2, NC_045512.2), *Brangacovirus* (Canada goose CoV Cambridge_Bay_2017, MK359255.1), *Cegacovirus* (Beluga Whale CoV SW1, NC_010646.1), *Igacovirus* (Turkey CoV, NC_010800.1), *Andecovirus* (Wigeon CoV HKU20, NC_016995.1), *Buldecovirus* (Common-moorhen CoV HKU21, NC_016996.1), *Herdecovirus* (Night-heron CoV HKU19, NC_016994.1)

Among all 8 suborders of the *Nidovirales* order, *Cornidovirineae* harbors the most epidemic viruses. Currently, the *Cornidovirineae* suborder contains 1 viral family, *Coronaviridae*, which is further divided into 2 subfamilies, *Letovirinae* and *Orthocoronavirinae* (Bukhari et al. 2018). The subfamily *Letovirinae* contains 1 genus *Alphaletovirus*, 1 subgenus *Milecovirus*, and 1 species *Microhyla letovirus* 1 (MLeV), which is represented by a 22.3 kb potentially partial genome found in an ornamented pygmy frog (*Microhyla fissipes*) (Bukhari et al. 2018). In contrast, the subfamily *Orthocoronavirinae* contains 4 genera, *Alphacoronavirus* (14 subgenera and 19 species), *Betacoronavirus* (5 subgenera and 14 species), *Deltacoronavirus* (3 subgenera and 7 species), and *Gammacoronavirus* (3 subgenera and 5 species), which is the largest subfamily in the *Nidovirales* order, including some highly pathogenic viruses, such as SARS-CoV, MERS-CoV, and SARS-CoV-2 (Table 1) (Coronaviridae Study Group of the International Committee on Taxonomy of Viruses 2020). In this review, “CoVs” refers to viruses of the subfamily *Orthocoronavirinae* (Figs. 2, 3, 4 and 5).

### Subfamily *Orthocoronavirinae*

CoVs are spherical, enveloped viral particles (approximately 100 nm in diameter) with S proteins on the surface. The subfamily *Orthocoronavirinae* is divided into 4 genera: *Alphacoronavirus* (Alpha-CoV), *Betacoronavirus* (Beta-CoV), *Deltacoronavirus* (Delta-CoV), and *Gammacoronavirus* (Gamma-CoV) (Wong et al. 2019). Alpha-CoVs and beta-CoVs infect mammals, while delta-CoVs and gamma-CoVs mostly infect birds, but a few of them do infect mammals (Woo et al. 2012). Currently, there are 14 subgenera (from *Colacovirus* to *Tegacovirus*), including 19 species in *Alphacoronavirus* with 5 subgenera (*Embecovirus*, *Hibecovirus*, *Mergecovirus*, *Nobecovirus* and *Sarbecovirus*), 14 species in *Betacoronavirus* with 3 subgenera (*Andecovirus*, *Buldecovirus* and *Herdecovirus*), 7 species in *Deltacoronavirus* with 3 subgenera (*Brangacovirus*, *Cegacovirus*, and *Igacovirus*), and 5 species in *Gammacoronavirus* (Table 1, Fig. 3).

### Alpha-CoVs

#### Human related alpha-CoVs

Alpha-CoVs contain 14 viral genera and 19 species and are the largest genus in the subfamily *Orthocoronavirinae* (Fig. 3). Two human CoVs (HCoVs) in alpha-CoVs, HCoV-229E and HCoV-NL63, which belong to subgenera *Duvinacovirus* and *Setracovirus*, respectively, have been identified (McIntosh et al. 1967; van der Hoek et al. 2004). These CoVs are prevalent worldwide in children and adults and cause respiratory infections, usually leading to mild cold-like symptoms and acute respiratory disease (El-Sahly et al. 2000; van der Hoek et al. 2004). Genetic evidence has shown that HCoV-229E and NL63 share common ancestors with bat CoVs, indicating the possibility of viral origin from bats (Tao et al. 2017). 229E-like bat CoVs, such as strains BtKY229E-1, BtKY229E-8 and BtCoV/FO1A-F2, have been detected in *Hipposideros* bats in Africa (Pfefferle et al. 2009; Crossley et al. 2012). Moreover, CoVs highly similar to 229E were also detected in camelids, including alpacas and dromedary camels (Crossley et al. 2012; Corman et al. 2016). The 229E-related CoVs isolated from dromedary camels can use human aminopeptidase N (APN) as a receptor and efficiently replicate in human hepatoma cells, which are neutralized by antibodies in human sera positive for 229E, suggesting a close genetic relationship between 229E and 229E-related CoVs (Corman et al. 2016). Due to the long-term contact and habitat overlap between humans and camels, one of the conjectures about the transmission path of 229E is that the ancestor bat CoV was transmitted to camels first and then from camels to humans (Corman et al. 2016). Bat and camel 229E-related CoVs have been designated into the same species represented by 229E because all of these CoVs share more than 95% amino acid (aa) sequence identity (higher than 92.4% according to the species demarcation criteria), with 229E in the concatenated protein domains of 3CLpro, NiRAN, RdRp, ZBD and HEL1 (Sabir et al. 2016; International Committee on Taxonomy of Viruses Executive 2020). NL63-like CoVs were also found in *Triaenops* bats, such as strains BtKYNL63-9a, BtKYNL63-9b, and BtKYNL63-15 (Tao et al. 2017). These NL63-like bat CoVs exhibited less than 90% similarity in their aa sequences compared to NL63 in the concatenated protein domains of 3CLpro, NiRAN, RdRp, ZBD and HEL1, and thus, they were classified into *NL63-related bat coronavirus strain BtKYNL63-9b* species, parallel to the *human coronavirus NL63* species in the subgenus *Setracovirus*. However, NL63-related CoVs have not yet been detected in livestock or other animals, so the origin and intermediate host(s) of NL63 are still unclear. Notably, NL63-related and 229E-related bat CoVs exhibit high genetic diversity in bats, and recombination of bat CoVs may accelerate the occurrence of new viruses and the possibility of cross-species transmission (Tao et al. 2017).

#### Bat alpha-CoVs and derived CoVs infecting other animals

Bats carry many different CoVs and have been recognized as important natural reservoirs for CoVs. Bat CoVs occupy 11 of 19 viral species in alpha-CoVs. The representative species of subgenus *Colacovirus* is *bat coronavirus CDPHE15*, which was identified in *Myotis lucifugus* (little brown bat) (Subudhi et al. 2017). The subgenus *Decacovirus* contains 2 species, *bat coronavirus HKU10* and *Rhinolophus ferrumequinum alphacoronavirus HuB-2013*, both of which were isolated from bats. HKU10- and HKU10-related CoVs were identified in 2 species of bats, *Rousettus leschenaultii* (Leschenault’s Rousettes) and *Hipposideros pomona* (Pomona leaf-nosed bat), which were named Ro-BatCoV HKU10 and Hi-BatCoV HKU10, respectively (Lau et al. 2012). Ro-BatCoV HKU10 and Hi-BatCoV HKU10 share high aa similarity (>99%) among 3CLpro, NiRAN, RdRp, ZBD and HEL1 but only 60.5% aa identity in the S proteins. Interspecies transmission of HKU10 from Leschenault’s Rousettes to Pomona leaf-nosed bats, 2 different bat suborders, has been reported in a previous study (Lau et al. 2012). The HKU10-related bat CoV was also identified in *Rhinolophus sinicus* (Rs-BatCoV HKU32), which may be a potential novel species (Lau et al. 2019b). The species of *Rhinolophus ferrumequinum alphacoronavirus HuB-2013* has several strains, among which the strain BtRf-Alpha-CoV/HuB2013 was detected in *Rhinolophus ferrumequinum*, and the strain BtMs- alpha-CoV/GS2013 was detected in *Myotis* bats (Wu et al. 2016). The subgenus *Minunacovirus* includes 2 species, *Miniopterus bat coronavirus 1* and *Miniopterus bat coronavirus HKU8*, which are common CoVs in *Miniopterus* bats, such as *Miniopterus schreibersii*, *Miniopterus fuliginosus* and *Miniopterus pusillus* (Chu et al. 2008; Wu et al. 2016).

The subgenus *Myotacovirus* contains 1 species, *Myotis ricketti alphacoronavirus Sax-2011*, which was found in *Myotis ricketti* in China in 2011 (Wu et al. 2016). The subgenus *Nyctacovirus* contains 2 species, *Nyctalus velutinus alphacoronavirus SC-2013* (BtNv-Alpha-CoV/SC2013) and *Pipistrellus kuhlii coronavirus 3398* (Bat-CoV/P.kuhlii/Italy/3398), which were detected in *Nyctalus* and *Pipistrellus* bats, respectively (Wu et al. 2016; De Sabato et al. 2019). Recently, bat CoV HKU33 (Tr-BatCoV HKU33) was discovered in *Tylonycteris robustula* (greater bamboo bats) in Guizhou Province in China (Lau et al. 2019b). Tr-BatCoV HKU33 is most closely related to BtNv-alpha-CoV/SC2013. However, in the concatenated protein domains, aa sequence identity is 76.3% between Tr-BatCoV HKU33 and BtNv-Alpha-CoV/SC2013, indicating that Tr-BatCoV HKU33 represents a novel CoV species and a potential novel subgenus (less than 14.7% homology with the 3CLpro, NiRAN, RdRp, ZBD and HEL1 domains) in the alpha-CoV genus (Lau et al. 2019b).

The subgenus *Pedacovirus* contains 2 species, PEDV and *Scotophilus bat coronavirus 512* (BtCoV/512). BtCoV/512 was discovered in *Scotophilus kuhlii* bats in Hainan, China (Tang et al. 2006). PEDV is the etiologic agent of porcine epidemic diarrhea (PED), which infects pigs of all ages and causes significantly high mortality in piglets under 7 days old with symptoms including diarrhea, vomiting, anorexia and dehydration (Chen et al. 2013). Since the recognition of PEDV in 1978 in Belgium, this virus has caused epidemics worldwide and has continuously impaired pig farming industry (Pensaert and Bouck 1978). During long-term epidemics, novel PEDV variants continuously emerge, deriving the virulent variants of SINDEL groups and different genotype groups including GI and GII (even more groups or subgenotypes) (Zeng et al. 2017). In addition, the coexistence or coinfection of multiple genotypes or variants of PEDV has been reported, which makes the prevention and control of PEDV more difficult (Zeng et al. 2017).

The subgenus *Rhinacovirus* has 1 species, *Rhinolophus bat coronavirus HKU2* (Bat-CoV HKU2). Bat-CoV HKU2 was first reported in 2006 and was detected in *Rhinolophus sinicus* (Chinese horseshoe bat) (Woo et al. 2006a). Subsequently, HKU2-like viruses were identified in other bat species, such as *Rhinolophus ferrumequinum* and *Rhinolophus affinis* (Lau et al. 2007; Wu et al. 2016). HKU2 is genetically distinct from other known bat alpha-CoVs and forms a unique phylogenetic lineage (Fig. 3) (Wu et al. 2016). Another remarkable feature of HKU2 is the shortest aa sequence of its S protein, which has deletions in the N-terminal region and extremely low (< 30%) aa identities with the other S proteins of CoVs (Lau et al. 2007). These significant differences indicate that HKU2 has a special evolutionary history. Because HKU2 has a long phylogenetic relationship with common alpha-CoV pathogens, it was not predicted to exhibit cross species transmission or lead to diseases until a similar virus was detected in piglets with diarrhea symptoms in China from 2016 to 2017 (Gong et al. 2017; Pan et al. 2017). In October 2016 and February 2017, outbreaks of newborn piglet diarrhea occurred in commercial pig farms in Guangdong Province, characterized by acute vomiting and watery diarrhea with a mortality rate over 35% in piglets (Pan et al. 2017). The etiology was identified as a Bat-HKU2 alpha-CoV called swine enteric alphacoronavirus (SeACoV), porcine enteric alphacoronavirus (PEAV), or SADS-CoV (Zhou et al. 2018). The genome sequence of SADS-CoV showed approximately 95% nucleotide (nt) identity with the Bat HKU2 strains (Pan et al. 2017). However, at the nt and aa levels, the S of SADS-CoV only exhibited approximately 80% and 86% sequence identities with that of bat HKU2 (Pan et al. 2017; Zhou et al. 2018). Recent studies showed that bat HKU2 strains derived from bats in Guangdong Province were genetically diverse in different *Rhinolophus* bats and exhibited different homologies to SADS-CoV, from 95.09% to 98.48% (Zhou et al. 2018). Virological, epidemiological, and evolutionary evidence suggested that SADS-CoV might have recently spread from bats to pigs and cause an outbreak of disease (Zhou et al. 2018). One study reported that SADS-CoV caused an epidemic across 4 farms, leading to the death of 24,693 piglets from October 2016 to January 2017 (Zhou et al. 2018). After that, new SADS cases were reported in Fujian Province of China in 2018 until January 2019 (Yang et al. 2020b) and in Southern China in February 2019 (Zhou et al. 2019). Moreover, experimental results in different laboratories showed that SADS-CoV has broad cell tropism and can infect cells derived from bats, mice, rats, gerbils, hamsters, pigs, chickens, nonhuman primates, humans and certain primary human cells, including different primary human lung cell types and primary human intestinal cells (Yang et al. 2019; Edwards et al. 2020; Luo et al. 2020). These results indicate that SADS-CoV already exhibits cross-species transmissibility from bats to pigs with the potential to spread to other animals. Although SADS-CoV was not identified after February 2019, it’s important to monitor SADS-CoV, bat HKU2 and similar CoVs for the prevention of disease outbreaks.

#### Alphacoronavirus 1

The subgenus *Tegacovirus* contains 1 viral species, *Alphacoronavirus 1*. This viral species includes a variety of common pathogens that infect livestock or pets (pigs, dogs, cats, etc.), such as TGEV, PRCV, CCoV and FCoV. In 1946, TGEV was first detected in the United States and is now endemic worldwide (He et al. 2020a). TGEV is a porcine enteropathogenic pathogen infecting pigs of all ages that leads to severe diarrhea and villous atrophy, causing high mortality in piglets (Doyle and Hutchings 1946). PRCV, which causes respiratory syndromes, is a variant derived from TGEV with a deletion in S gene (Wang and Zhang 2017). CCoV is a worldwide gastroenteric pathogen causing mild enteritis in young dogs (Decaro et al. 2012). CCoV is divided into 2 subtypes, CCoV type I (CCoV-I) and type II (CCoV-II), based on genetic divergence in S gene (Pratelli et al. 2003). FCoV infects cats and is endemic in different regions around the world. Normally, FCoV infection is asymptomatic or causes mild enteric disease, but some cases (approximately 5 %) lead to feline infectious peritonitis (some mutant FCoVs are also known as feline infectious peritonitis virus, FIPV) (Lewis et al. 2015). CoVs of *Alphacoronavirus 1*, including TGEV, PRCV, CCoV and FCoV, are closely related. Homologous recombination occasionally occurs in these viruses, leading to the emergence of new strains. For instance, the CCoV-IIb subtype of CCoV-II is a recombinant form of CCoV-II and TGEV, and serotype 2 of FCoV was generated *via* the recombination of type 1 FCoV and CCoV (Herrewegh et al. 1998; Decaro et al. 2009). CoVs high similar to *Alphacoronavirus* *1* have not been detected in wild animals, such as bats, and thus, the exact origin of these viruses is unclear.

#### Other alpha-CoVs

The subgenus *Minacovirus* has 1 species, *mink coronavirus 1* (MCoV). MCoV causes epizootic catarrhal gastroenteritis (ECG) in mink with high morbidity (approximately 100 %) and low mortality (<5 %) and has affected mink industry in Europe, America and Asia (Gorham et al. 1990). ECG disease was first reported in 1975, and CoV-like particles were identified in ECG biopsies by electron microscopy in the 1990s; however, the genome of MCoV was not identified until the 2010s (Gorham et al. 1990; Vlasova et al. 2011). In 2011, Anastasia et al. reported the full-length genome sequences of 2 MCoVs (WD1127 from Wisconsin and WD1133 from Minnesota), sharing 91.7% nt identity between their genomes (Vlasova et al. 2011). WD1127 and WD1133 share high aa identity in ORF1a and ORF1b polyproteins (94.2% and 97.9%, respectively) but only 86.3% aa identity in S proteins. In addition to MCoV, 2 types of ferret coronavirus (FRCoV), ferret systemic coronavirus (FRSCV) and ferret enteric coronavirus (FRECV), were reported (Wise et al. 2010). FRECV infection of ferrets does not cause high mortality rates, but FRSCV infection can lead to systemic pyogranulomatous inflammation and may be fatal (Wise et al. 2006). Overall, the aa identity between FRCoV and MCoV is approximately 90% in the conserved replicase domains, suggesting that FRCoV and MCoV may represent 2 different species based on the latest classification standard (Lamers et al. 2016; International Committee on Taxonomy of Viruses Executive 2020).

The subgenus *Luchacovirus* has 1 species, *Lucheng Rn rat coronavirus* (RatCoV LRNV). LRNV was detected in *Rattus norvegicus* samples collected in 2013 in China (Wang et al. 2015). Recently, additional CoVs have been identified in rats and voles, including the AcCoV-JC34 strain in *Apodemus chevrieri*, the RtMruf-CoV-1/JL2014 strain in *Myodes rufocanus*, the RtRl-CoV/FJ2015 strain in *Rattus losea* and the UKRn3 strain in *Rattus norvegicus* (Ge et al. 2017; Wu et al. 2018; Tsoleridis et al. 2019). These rodent CoVs share greater than 90% aa identity in the conserved replicase domains but had less than 70% aa identity in S proteins, which may reflect their adaptation in different hosts. Usually, all rodent CoVs are clustered together in phylogenetic trees based on different genes, which indicates a common ancestor of them (Tsoleridis et al. 2019). In addition, 3 other strains detected in Europe, L232 in Oryctolagus cuniculus (rabbit) in France, UKMa1 in *Microtus agrestis* (field vole) in the United Kingdom and PLMg1 in *Myodes glareolus* (bank vole) in Poland were phylogenetically clustered together with rodent alpha-CoVs in the ORF1b tree but showed a certain genetic distance from other rodent alpha-CoVs (Tsoleridis et al. 2019). Surprisingly, rodent alpha-CoVs cluster together with HKU2, including SADS-CoV and other related bat CoVs, in the beta-CoV group based on the spike tree, which indicates an ancient recombination event between them (Pan et al. 2017; Tsoleridis et al. 2019). Rodentia is the largest mammalian order, harboring ~2,200 species. Rodents carry diverse pathogens and are an important source of emerging viral infections. Recently, an increasing number of CoVs have been isolated from rodents, and more are expected in the future. Since rodents and many kinds of animals share habitats, monitoring rodent CoVs could be beneficial for the prevention and control of CoV-related diseases.

The subgenera *Soracovirus* and *Sunacoviru*s each contain 1 viral species, *Sorex araneus coronavirus T14* (Shrew-CoV/Tibet2014) and *Suncus murinus coronavirus X74* (Xīngguō-74 or known as WESV), respectively. Shrew-CoV/Tibet2014 and Xīngguō-74 have been identified in different shrews, *Sorex araneus* and *Suncus murinus*, respectively, in China (Wang et al. 2017; Wu et al. 2018). Both of them belong to the *Soricidae* family and *Soricomorpha* order. In addition to Xīngguō-74, other strains were also identified in shrews in China, including Wénchéng shrew virus (WESV) and Yúdū shrew virus (Yúdū-76), which showed moderate genetic diversity (Wang et al. 2017). CoVs detected in shrews in China, which are represented by Shrew-CoV/Tibet2014 and Xīngguō-74, are clustered together, forming a distinct group. However, shrew CoVs show distinct phylogenetic relationships to other known alpha-CoVs, indicating that these viruses are from different origins and have distinct evolutionary characteristics (Wang et al. 2017; Wu et al. 2018).

### Beta-CoVs

The genus *Betacoronavirus* currently contains 5 subgenera (*Embecovirus*, *Hibecovirus*, *Mergecovirus*, *Nobecovirus*, and *Sarbecovirus*) and 14 species. Beta-CoV is well known because it includes the most pathogenic CoVs to human beings, such as SARS-CoV, MERS-CoV and SARS-CoV-2, as well as a large number of related bat CoVs (Hu et al. 2015; Wong et al. 2019; Hu et al. 2021). The Beta-CoV species include *Betacoronavirus 1*, *China rattus coronavirus HKU24*, *human coronavirus HKU1*, *murine coronavirus* and *Myodes coronavirus 2JL14* in subgenus *Embecovirus*, *bat Hp-betacoronavirus Zhejiang2013* in subgenus *hibecovirus*, *hedgehog coronavirus 1*, *Middle East respiratory syndrome-related coronavirus*, *pipistrellus bat coronavirus HKU5* and t*ylonycteris bat coronavirus HKU4* in subgenus *Merbecovirus*. *Eidolon bat coronavirus C704*, *Rousettus bat coronavirus GCCDC1*and *Rousettus bat coronavirus HKU9* in subgenus *Nobecovirus*, and *severe acute respiratory syndrome-related coronavirus* in subgenus *Sarbecovirus*.

#### Human beta-CoVs

##### HCoV-OC43 and HKU1

Five types of beta-CoVs classified into 4 representative species have been reported to infect humans: HCoV-OC43 of *Betacoronavirus 1*, HCoV-HKU1, MERS-CoV of *Middle East respiratory syndrome-related coronavirus* (MERSr-CoV), SARS-CoV and SARS-CoV-2 of *severe acute respiratory syndrome-related coronavirus* (SARSr-CoV) (Hu et al. 2021). OC43 and HKU1 were discovered in patients with respiratory diseases in 1967 and 2005, respectively (Hamre and Procknow 1966; Woo et al. 2005). OC43 and HKU1, together with the other 2 alpha-CoVs, 299E and NL63, are associated with a wide range of respiratory outcomes, including bronchiolitis and pneumonia and cause nonrespiratory organ system diseases, such as enteric and nervous systems (Zeng et al. 2018). Although these CoVs are considered one of the most common respiratory pathogens associated with respiratory tract infections in children and elderly individuals, particularly severe infections in infants, clinical epidemiological investigations have revealed a higher positive rate of these CoVs in adults, ranging from 50 to 59 years old (Cabeça et al. 2013). In some seasons, OC43 could be the most frequent virus detected in acute respiratory tract infection cases and had a more frequently abnormal pulmonary rate than the other 3 CoVs (Zeng et al. 2018).

OC43 has a global distribution, and circulating OC43 viruses have high genetic diversity with at least 5 distinct genotypes (A to E) (Kin et al. 2015). Recombination of different types promotes the generation of new viral types. For example, OC43-D emerged *via* recombination between genotypes B and C, and OC43-E emerged *via* recombination among genotypes B, C, and D (Zhang et al. 2015). In viral classification, OC43 belongs to the species *Betacoronavirus 1*, together with various closely related CoVs detected in other animals (Fig. 3). In addition to OC43, the other related CoVs in species *Betacoronavirus 1* include HKU23 detected in dromedary camels, bovine CoV (BCoV) in bovine and alpaca, SACoV in *Sable antelope*, GiCoV in giraffe, canine respiratory CoV (CRCoV) in dog, porcine hemagglutinating encephalomyelitis virus (PHEV) and equine CoV (ECoV) (Fig. 3) (Woo et al. 2014b). Phylogenetically, CoVs of *betacoronavirus 1* are most closely related to another species, *China Rattus coronavirus HKU24* (HKU24), in the subgenus *Embecovirus* (Fig. 3). HKU24 and similar viruses have been found in rats, such as *Rattus norvegicus*, *Apodemus agrarius*, and *Apodemus chevrieri* (Lau et al. 2015; Wang et al. 2015; Ge et al. 2017). The RdRp and Hel proteins of OC43 and HKU24 exhibit 91.8% and 93.5% aa sequence identity, respectively (Lau et al. 2015). Current evidence indicates that OC43 likely originated from rodents and may be transmitted to humans *via* livestock, such as bovines (Wang et al. 2015; Forni et al. 2017; Ge et al. 2017).

HKU1 was first reported in 2005 in a 71-year-old patient with pneumonia and bronchiolitis in Hong Kong (Woo et al. 2005). Since then, HKU1 has been detected in France, USA, Brazil, Australia, etc., indicating its global distribution (Siu et al. 2014; Zeng et al. 2018). Strains of HKU1 are divided into 3 genotypes, A, B and C, based on their phylogenetic relationships (Woo et al. 2006b). Recombination between different genotypes may lead to the emergence of novel genotypes. For instance, the recombination of hemagglutinin esterase (HE) coding regions between A and B generated genotype C (Woo et al. 2006b; Dominguez et al. 2014). HKU1 is closest to *murine coronavirus* (known as mouse hepatitis virus, MHV) according to phylogenetic analysis, and different strains of *murine coronavirus* have been detected in rats (rat CoV Parker) and mice (MHV, Fig. 3) (Das Sarma et al. 2001). One hypothesis is that HKU1 originates from rodents, but its intermediate host is still unclear (Forni et al. 2017; Cui et al. 2019).

##### SARS-CoV and SARS-CoV-2

Severe acute respiratory syndrome coronavirus (SARS-CoV) and severe acute respiratory syndrome coronavirus 2 (SARS-CoV-2) are highly pathogenic human CoVs (Falsey and Walsh 2003; Zhu et al. 2020). SARS-CoV caused a relatively short epidemic of SARS in 2002–2003, resulting in more than 8,000 clinical cases with a mortality of 10% (Parry 2003; Stadler et al. 2003). However, ongoing SARS-CoV-2 has caused an extensive pandemic, termed coronavirus disease 2019 (COVID-19), beginning the end of 2019, leading to more than a hundred million cases and millions of deaths worldwide (Hu et al. 2021). Both SARS-CoV and SARS-CoV-2 are primarily transmitted and enter through the respiratory tract, and the primary symptom is acute severe pneumonia (Huang et al. 2020a; Zhu et al. 2020). However, compared to SARS-CoV, SARS-CoV-2 reportedly has a wider histotropic range of infection, including the intestinal tract, kidney, nervous system and so on (Chen et al. 2020a; Hu et al. 2021; Huang et al. 2020a). In addition, SARS-CoV-2 exhibits high transmissibility, with an R0 value estimated as 2.3 but that could be as high as 5.7, and a long incubation period of 5.4 median incubation days is estimated for SARS-CoV-2 infection, which can be as long as 13.7 days in 95% of symptomatic cases and even longer in some asymptomatic cases (Yang et al. 2020a). The COVID-19 pandemic has become a serious global public health problem that has challenged our knowledge of virology and systems of viral disease control.

The overall genomic sequence identity between SARS-CoV and SARS-CoV-2 is 79.6%; however, aa identities of ORF1a and ORF1b between them are 80.9% and 95.7%, respectively (Zhou et al. 2020b). Based on the new demarcation criteria, they belong to the same species, *severe acute respiratory syndrome-related coronavirus* (SARSr-CoV) Coronaviridae Study Group of the International Committee on Taxonomy of Viruses 2020). In CoV taxonomy, SARS-CoV, SARS-CoV-2, most bat CoVs and a few CoVs detected in civets, pangolins and other animals are classified in the SARSr-CoV subgenus *Sarbecovirus* (Fig. 4) (Coronaviridae Study Group of the International Committee on Taxonomy of Viruses 2020). Since the appearance of SARS, investigations on the origin of SARS-CoV have led to the discovery of a large number of novel CoVs in various animals, particularly in bats, and greatly promoted the understanding of the existence and spread of CoVs in nature (Hu et al. 2015; Wong et al. 2019). In 2005, CoV strains highly homologous to SARS-CoV were identified in the Chinese horseshoe bat (*Rhinolophus sp.* ), including Rp3, HKU3, etc. (Li et al. 2005; Woo et al. 2005). SARS-CoV uses angiotensin-converting enzyme 2 (ACE2) as its receptor to enter cells (Li et al. 2003). In 2013, WIV1, a living bat CoV using the same ACE2 receptor as SARS-CoV, was isolated from a *Rhinolophus sinicus* sample (Li et al. 2003; Ge et al. 2013). The overall nt identity of WIV1 genome with human SARS-CoV is 95.4%. In 2015, another bat CoV strain, WIV16, which shows higher genomic sequence identity (96% nt identities) to SARS-CoV, was isolated (Yang et al. 2015). A 5-year surveillance of bat SARSr-CoVs in Yunnan Province, China, revealed that the SARSr-CoVs circulating in bats are highly diverse and have high genetic similarity to SARS-CoV in the hypervariable N-terminal domain (NTD) and receptor-binding domain (RBD) of S1 gene, ORF3 and ORF8, respectively (Hu et al. 2017). During the SARS epidemic, SARS-CoV was detected in some animal species, such as civets in southern China, which showed genomic nt identities of 99.8% to human SARS-CoV, indicating interspecies transmission of SARS-CoV between animals and humans (Guan et al. 2003).

Benefitting from previous SARS-CoV and SARSr-CoV research, within a short time after the outbreak of SARS-CoV-2, a bat CoV strain (RaTG13) showing high similarity to SARS-CoV-2 was identified (Zhou et al. 2020b). The overall nt identity of genomes between SARS-CoV-2 and RaTG13 is 96.2%, and the aa identities of ORF1a, ORF1b, and S protein between SARS-CoV-2 and RaTG13 are 98%, 99.3%, and 97.7%, respectively, suggesting that SARS-CoV-2 and RaTG13 are highly homologous (Zhou et al. 2020b). Although the aa identity of S proteins between SARS-CoV and SARS-CoV-2 is only approximately 77%, SARS-CoV-2 also uses ACE2 as its entry receptor (Zhou et al. 2020b). Other CoV strains close to SARS-CoV-2 include bat SARSr-CoV ZC45, ZXC21 and RmYN02 (Fig. 4) (Hu et al. 2018; Zhou et al. 2020a, b), but their genomes exhibit lower homology to that of SARS-CoV-2 than RaTG13. It is worth noting that RaTG13, ZC45/ZXC21 and RmYN02 were found in different *Rhinolophus* bat species: *Rhinolophus affinis*, *Rhinolophus pusillus*, and *Rhinolophus malayanus*, respectively. In addition to bats, CoV strains (pangolin-CoV) close to SARS-CoV-2 were also detected in pangolins (Lam et al. 2020; Xiao et al. 2020). Pangolins infected with pangolin-CoV showed clinical signs and histological changes, indicating that pangolins are probably not a natural reservoir but an intermediate host of SARS-CoV-2 (Xiao et al. 2020). However, all SARSr-CoVs detected in bats (RaTG13, ZC45/ZXC21, and RmYN02) and pangolins (pangolin-CoV) are different from human SARS-CoV-2 in their genomic sequences and certain special genes. In particular, S protein of SARS-CoV-2 harbors a unique furin cleavage site that is lacking in animal SARSr-CoVs, suggesting that SARSr-CoVs are not the origin of SARS-CoV-2 (Wang et al. 2020b). Due to the long-term pandemic of SARS-CoV-2, it has been detected in a wide variety of animals, including cats, tigers, minks and dogs, but they are likely to be transmitted from COVID-19 patients (Oude Munnink et al. 2021). Both SARS-CoV and SARS-CoV-2 exhibited broad ACE2 usage, indicating their wide range of hosts (Wang et al. 2020a).

In the phylogenetic tree based on S proteins, SARSr-CoV formed 3 clusters. Human and civet SARS-CoV strains are clustered with various bat SARSr-CoVs derived from *Rhinolophus sinicus*, *Rhinolophus pusillus*, *Rhinolophus ferrumequinum*, *Rhinolophus macrotis*, etc. However, the most homologous strains were all detected from *Rhinolophus sinicus*, such as WIV1, indicating the host specificity of these CoVs. The SARS-CoV-2 strains cluster with RaTG13, pangolin-CoV, ZC45/ZXC21 and RmYN02. Moreover, the other 2 bat SARSr-CoVs, JL2012 and Rf4092, cluster together. These results suggest that *Rhinolophus* bats are a natural reservoir of SARSr-CoVs and bat SARSr-CoVs occasionally spill over across species to infect humans or other animals. However, the exact timing of the spillover of SARSr-CoVs remains obscure.

##### MERS-CoV

MERS-CoV is detected in both humans and dromedaries. Several closely related bat CoVs are classified into the species *Middle East respiratory syndrome-related coronavirus* in the subgenus *Merbecovirus* (Wong et al. 2019). Symptoms caused by MERS-CoV infection range from none to mild or severe respiratory ailments, including fever, cough, shortness of breath, and, on occasion, pneumonia and gastrointestinal symptoms (de Wit et al. 2016). Since the first report of MERS in Saudi Arabia in 2012, 2519 laboratory-confirmed cases of MERS-CoV have been reported, causing 866 deaths (34.3% mortality) in 27 countries globally (Zaki et al. 2012; http://www.emro.who.int/pandemic-epidemic-diseases/mers-cov/mers-situation-update-january-2020.html). To date, MERS cases have been reported in 12 countries in the Middle East and are prevalent primarily in Saudi Arabia, where 80% of all MERS cases have been reported (Chafekar and Fielding 2018). MERS cases have also been reported in countries outside the Middle East, likely due to international travel (Fanoy et al. 2014). Unlike SARS-CoV and SARS-CoV-2, MERS-CoV exhibits limited human-to-human transmission and does not usually cause sustainable epidemics (Mailles et al. 2013). However, an explosive MERS epidemic was reported in Korea from 20 May to 13 June 2015, in which an imported MERS case led to 185 infections and 36 deaths (Korean Society of Infectious Diseases and Korean Society for Healthcare-associated Infection Control and Prevention 2015).

MERS-CoV is a zoonotic virus, and transmission of this virus from dromedaries to humans through direct or indirect contact with infected animals has been documented (The Health Protection Agency Uk Novel Coronavirus Investigation Team 2013; Memish et al. 2014). CoVs isolated from dromedaries showed >99% genomic nt identity with human MERS-CoV, further demonstrating transmission of the virus between camels and humans (Chu et al. 2014; Haagmans et al. 2014). Nucleic acid detection, sequencing and phylogenetic evidence indicates that MERS-CoV exists in dromedaries in Africa and the Middle East, confirming that the virus is prevalent in dromedaries (Reusken et al. 2014; Sabir et al. 2016). In addition, serological evidence on MERS-CoV revealed that antibodies in dromedaries in Africa and the Middle East exhibit high neutralizing titers against MERS-CoV (Reusken et al. 2013; Ommeh et al. 2018). Antibodies against MERS-CoV in dromedaries were detected as early as the 1980s, indicating the long-term existence and infection of MERS-CoV or MERSr-CoV in dromedaries (Müller et al. 2014). As a result, the exact time in history when MERS-CoV spread between camels and humans and among humans remains uncertain.

In bats, a few strains of MERSr-CoV have been detected. In 2014, a bat CoV detected in the South African *Neoromicia capensis* bat (NeoCoV) was clustered as a basal sister clade of MERS-CoV. NeoCoV had 92.7%, 96.4%, 98.6% and 98.5% aa sequence identities with ORF1ab polyprotein, 3CLpro, RdRp and Hel proteins of MERS-CoV, respectively (Corman et al. 2014a). In addition to NeoCoV, bat MERSr-CoVs were also discovered in *Pipistrellus* bats in Romania and Ukraine, *Eptesicus* bats in Italy, *Nyctinomops* bats in Mexico, *Vespertilio superans* bats in China (BtVs-BetaCoV/SC2013), *Pipistrelle* bats in China (HKU25), etc., and these bat MERSr-CoVs share more than 96% aa identity with MERS-CoV in the partial RdRp protein (Yang et al. 2014a; Hu et al. 2015; Lau et al. 2018b). Therefore, these bat CoVs and MERS-CoV originated form the MERSr-CoV species. Nevertheless, bat MERSr-CoVs are unlikely to be the direct ancestor of MERS-CoV due to the extremely low similarity in some genes between bat MERSr-CoVs and MERS-CoV. For example, NeoCoV and MERS-CoV share 64.3-64.6% aa identity only in S proteins (Corman et al. 2014a). The transmission of MERSr-CoVs among bats, dromedaries and humans remains to be further revealed.

In the subgenus *Merbecovirus*, there are 3 other viral species, *Hedgehog coronavirus 1* (HedCoV1), *Tylonycteris bat coronavirus HKU4* (HKU4), and *Pipistrellus bat coronavirus HKU5* (HKU5) (Lau et al. 2019a). After the emergence of MERS-CoV, HedCoV1 was identified in hedgehogs in Europe and China and named EriCoV and Ea-HedCoV HKU31 (HKU31), respectively (Corman et al. 2014b; Lau et al. 2019a). EriCoV and HKU31 are classified in HedCoV1 species, which is the species closest to MERSr-CoV according to their genomic similarity and phylogenetic relationship (Lau et al. 2019a). Before the emergence of MERS-CoV in 2007, HKU4 and HKU5 were detected in lesser bamboo bats (*Tylonycteris pachypus*) and Japanese pipistrelle bats (*Pipistrellus abramus*), respectively (Woo et al. 2007). HKU4 and HKU5 came form 2 independent sister species, which are close to the other two species, HedCoV1 and MERSr-CoV (Fig. 3) (Lau et al. 2018b). Evidence that bat CoVs, such as HKU4 and HKU25, use human dipeptidyl peptidase 4 (hDPP4), the MERS-CoV receptor, for cell entry suggests a bat origin for MERS-CoV (Yang et al. 2014b; Lu et al. 2015; Lau et al. 2021).

#### Other bat beta-CoVs

In addition to bat CoVs HKU4, HKU5, MERSr-CoV, and SARSr-CoV mentioned above, there are 4 other species of bat beta-CoVs, including *bat Hp-betacoronavirus Zhejiang2013* (BtHp-BetaCoV/ZJ2013) in the subgenus *Hibecovirus*, *Eidolon bat coronavirus C704* (CMR704), *Rousettus bat coronavirus GCCDC1* (Ro-BatCoV GCCDC1), and *Rousettus bat coronavirus HKU9* (HKU9) in the subgenus *Nobecovirus*. BtHp-betaCoV/ZJ2013 has been observed in *Hipposideros pratti* bats in China (Wu et al. 2016). BtHp-BetaCoV/ZJ2013 showed 78% RdRp aa identity with beta-CoVs and represents a separate clade in the tree of beta-CoVs (Fig. 3). BtHp-betaCoV/ZJ2013 also displays a unique genomic structure containing an ORF2 between ORF1ab and S gene (Fig. 5) (Wu et al. 2016). CMR704 was detected in *Eidolon helvum* bats in Cameroon (Yinda et al. 2018). Ro-BatCoV GCCDC1 was first identified in *Rousettus leschenaulti* bats in Yunnan, China in 2016. The most remarkable feature of the Ro-BatCoV GCCDC1 genome is a 3′-end derived from reovirus, indicating a cross-family recombination event between CoV and reovirus (Huang et al. 2016). Similar recombination was also reported in a CoV detected in lesser dawn bats (*Eonycteris spelaea*) in Singapore (Paskey et al. 2020). HKU9 was first identified in Leschenault’s rousette bats (*Rousettus lechenaulti*) in China and has been commonly observed in *Rousettus* bats (Woo et al. 2007). It is worth mentioning that BtHp-BetaCoV/ZJ2013, Ro-BatCoV GCCDC1, and HKU9 in the subgenus *Nobecovirus* all form a distinct branch in the tree and are detected in fruit bats only, indicating the host specificity of these viruses (Fig. 3).

#### Rodent beta-CoVs

All rodent beta-CoVs are classified in the subgenus *Embecovirus*. In addition to HKU24 and *murine coronavirus* (MHV) mentioned above, another rodent viral species, *Myodes coronavirus 2JL14* (RtMruf-CoV-2/JL2014), is in the subgenus *Embecovirus* (Wu et al. 2018). *2JL14* was detected in gray-sided voles (*Myodes rufocanus*) in China in 2018 (Wu et al. 2018). Similar to other rodent beta-CoVs, it’s also close to CoVs in the species *Betacoronavirus 1* in the subgenus *Embecovirus* (Fig. 3).

### Gamma-CoVs

The genus *Gammacoronavirus* consists of 3 subgenera: *Igacovirus*, *Brangacovirus* and *Cegacovirus* (Lefkowitz et al. 2018). *Igacovirus* subgenus contains 3 species*: avian coronavirus*, *avian coronavirus 9203*, and *duck coronavirus 2714*. Viruses of *Igacovirus* subgenus primarily infect birds, such as chickens (*Gallus gallus*), turkeys, ducks, geese, pheasants, partridges, pigeons and peafowl (Cavanagh et al. 2002; Jonassen et al. 2005; Cavanagh 2007; Sun et al. 2007; Domanska-Blicharz et al. 2020). Infectious bronchitis virus (IBV) is the prototype virus of *Igacovirus* subgenus, and various serotypes of IBV have been identified. IBV is the causal agent of infectious bronchitis that is prevalent worldwide and primarily infects the upper respiratory tract, but several IBV serotypes also infect kidney, digestive tract and reproductive system (Reddy et al. 2015; Hou et al. 2020). The genome of IBV contains at least 10 ORFs characterized by the following organization: 5’UTR-ORF1a/1b-S-3a-3b-E-M-5a-5b-N-3’UTR. High rates of mutation and recombination have been observed in IBV genome and are considered to be the key factor driving adaptive evolution and host shift in this virus (Hewson et al. 2011; Latinne et al. 2020; Marandino et al. 2017). Genomic mutations of IBV are primarily located in the coding region of S protein, among which some mutations are reported to reduce the virulence of IBV, and most adaptive marker mutations occur in S1 subunit (Cavanagh et al. 1988). Nevertheless, mutations of S2 subunit have been reported to alter the membrane fusion ability of viral particles, leading to potential infectivity in nonbird species, such as mammals (Fang et al. 2005). In addition to S protein, coding regions of ORF1ab, especially those of NSP2, NSP3 and NSP16, also harbor many hotspots for mutation and recombination (Thor et al. 2011). Similar to mutations, most recombination events of IBV genome also tend to reduce the virulence of IBV (Huang et al. 2020b).

Subgenus *Brangacovirus* contains only 1 species, *goose coronavirus CB17*, which is named after the newly identified gammacoronavirus Canada goose coronavirus (CGCoV) that infects the goose species *Branta canadensis* (Papineau et al. 2019). Genome of CGCoV aligns with the following structure: 5’UTR-ORF1a/1b-S-ORF3-4a-E-M-5b-ORF6-7a-7b-8a-8b-N-ORF10-ORF11-3’UTR. Similar to avian coronaviruses (ACoVs), several transcription regulatory sequences (TRSs) are located at the end of each leader sequence of CGCoV, but the number of TRSs in CGCoV is twice as many as that of ACoVs (Papineau et al. 2019).

Subgenus *Cegacovirus* is represented by 2 viral strains, beluga whale coronavirus SW1 (BWCoV SW1) and bottlenose dolphin coronavirus HKU22 (BdCoV HKU22), which infect marine mammals (Mihindukulasuriya et al. 2008; Woo et al. 2014a). BWCoV SW1 belongs to the species of *beluga whale coronavirus SW1* characterized by the genomic structure 5’UTR-ORF1a/1b-S-E-M-5a-5b-5c-ORF6-ORF7-ORF8-ORF9-ORF10-N-3’UTR. Comparative genomic analyses demonstrated that BdCoV HKU22 harbors the same genomic structure as BWCOV SW1, particularly with respect to the putative TRSs, indicating that these 2 viruses can be classified into 1 species; however, this classification is still controversial since they share only 74.3-74.7% aa identity on their spike genes (Woo et al. 2014a). Currently, it is widely believed that BWCoV SW1 and BdCoV HKU22 both evolved from ACoVs (Woo et al. 2014a).

Gamma-CoVs do not express active NSP1, such as alpha-CoVs and beta-CoVs, to inhibit host protein production due to a lack of cleavage sites between NSP1 and NSP2. Alternatively, IBV expresses 5b protein, performing similar functions to NSP1 of other coronaviruses (Kint et al. 2016). Among the subgenera of gamma-CoVs, *Brangacovirus* and *Cegacovirus* lack the homologs of accessory proteins 3a and 3b, which are expressed by *Igacovirus* (Papineau et al. 2019), indicating high genomic similarity between *Brangacovirus* and *Cegacovirus* but not *Igacovirus* (Fig. 5). A recent study indicated that *Brangacovirus* is likely a transition stage in the evolution from *Igacovirus* to *Cegacovirus* (Papineau et al. 2019).

### Delta-CoVs

There are 3 subgenera (*Andecovirus*, *Buldecovirus*, and *Herdecovirus*) in the genus *Deltacoronavirus*, harboring CoVs infecting mammals (Asiana leopard cat, Chinese ferret badger, and porcine) and birds (bulbul, thrush, munia, white eye, sparrow, magpie robin, night heron, wigeon, common moorhen, falcon, houbara, pigeon, and quail) (Dong et al. 2007; Woo et al. 2009; Woo et al. 2012; Lau et al. 2018a; Wang et al. 2021). Among mammalian delta-CoVs, porcine deltacoronavirus (PDCoV) has become one of the primary pathogens causing pig diarrhea since 2014 (Li et al. 2014; Wang et al. 2014a). PDCoV was first discovered in a surveillance study in Hong Kong in 2012, and 2 strains with completed genomes were named HKU15-44 and HKU15-155 (Woo et al. 2012). Subsequently, PDCoVs were reported in other countries, including the United States, South Korea, Japan, mainland China, Thailand, Laos, Vietnam and other regions (Lee and Lee 2014; Li et al. 2014; Song et al. 2015; Janetanakit et al. 2016; Lorsirigool et al. 2016; Le et al. 2018; Suzuki et al. 2018).

PDCoV infects the digestive tracts of pigs, resulting in acute diffuse, severe atrophic enteritis with vomiting, watery diarrhea and severe dehydration with similar clinical symptoms to PED or TGE (Zhang 2016). The mortality of PDCoV-infected pigs ranges from 30 to 50% in piglets, which has seriously threatened pig breeding industry (Zhang 2016). Porcine aminopeptidase N (pAPN) has been demonstrated to be a functional receptor sufficiently mediating PDCoV entry (Wang et al. 2018). However, either knockout of pAPN or pAPN-specific antibody treatment cannot block the entry of PDCoV, indicating the existence of unknown redundant receptor(s) for PDCoV (Zhu et al. 2018). In addition, PDCoV may induce cellular autophagy (Qin et al. 2019).

The typical genomic organization of PDCoV is 5’UTR-ORF1a/1b-S-E-M-ORF6-N-7a/b/c-3’UTR, and the complete genome nucleotide identity of PDCoV strains in different regions ranges from 97.1% to 99.9%, indicating a single genotype of PDCoV around the world (Zhang 2016). PDCoV can be divided into 4 genetic lineages: the United States-Japan-South Korea lineage, the Chinese lineage, the Thailand-Laos-Vietnam lineage and the early Chinese lineage (He et al. 2020b). Each lineage is relatively evolutionarily independent, while a recent study found that PDCoV strains SD2018/10 and AH2019/H in China originated from the United States-Japan-South Korea lineage (He et al. 2020b).

Currently, there is still no direct evidence showing that PDCoV is transmitted by birds, and significant recombination events were only observed in porcine or bird *deltacoronavirus* (Lau et al. 2018a; Zhang et al. 2019; He et al. 2020b). However, it’s generally believed that PDCoV and Asian leopard cat coronavirus (AlCCoV) evolved from bird coronavirus, and the closest evolutionary species to PDCoV among bird coronaviruses is the sparrow *deltacoronavirus* HKU17 (SpDCoV-HKU17) (Woo et al. 2012). It’s worth noting that SpDCoV-HKU17 in the United States shares approximately 83.9% whole-genome nucleotide identity and greater than 85% aa identity for S protein with PDCoV, which provides more evidence for the bird origin of PDCoV (Chen et al. 2018).

Recently, 3 Haitian children with acute undifferentiated febrile illness were tested positive for PDCoV (https://doi.org/10.1101/2021.03.19.21253391), which is the first report of delta-coronavirus infecting humans. Considering the close contact of people with pigs and other domestic animals, the potential of cross-species transmission of delta-coronaviruses to humans should not be neglected. We must be wary of the evolution of these virus in mammals to prevent related epidemics in the future. 

### Genome structure of CoVs

Genomic sizes of the known CoVs ranged from 24.5 kb to 31.8 kb. Generally, genome size of CoVs in different genera varies; however, it tends to be similar in the same genus (Fig. 5). Genome length of beta-CoVs is similar, and the average size is approximately 30 kb. Delta-CoVs have the shortest genome length, which is approximately 25 kb. However, regardless genome size, genomic structure of CoVs is quite similar. genome organization follows the rule 5’-leader-UTR-replicase-S-E-M-N-3’-UTR-poly(A) tail, with accessory genes interspersed within genes and the 3’ end of genome (Figs. 2 and 5) (Lu et al. 2020). The first two-thirds of genome encodes ORF1a and ORF1b polyproteins by ribosomal frame shifting mode, and polyproteins are cut into multiple proteins by a protease of virus itself, which contains unique protease activities (Posthuma et al. 2008). The cleaved proteins are nonstructural polyprotein 1-16 (nsp1-16), including RdRp, Hel, 3CLpro and other important domains that perform multiple functions in the process of genomic replication (Fig. 2a) (Nukoolkarn et al. 2008). Structural proteins of S, E, M and N are responsible for the formation of viral structures (Fig. 2b). In particular, S protein is a type of surface glycoprotein responsible for binding and recognizing host cell receptors, coordinating viral entry, and determining host tropism of virus to some extent (Gui et al. 2017). Other accessory genes with interspersed distribution, such as ORF3, ORF4, ORF5 and eta, are also redundant genes. However, evidence showed that these genes do have specific functions, such as regulating host immune response and potentially promoting virus replication (Li et al. 2020b).

Although the overall structure of CoV genomes is relatively consistent, there are some detailed differences among different viruses. For example, bat CoV genome of BtHp-BetaCoV/ZJ2013 in subgenus *Hibecovirus* in genus *Betacoroanvirus* contains an ORF2 between ORF1ab and gene (Fig. 5) (Wu et al. 2016). CoVs in the subgenus *Embecovirus* in the genus *Betacoroanvirus* contain ORF2 and an HE between ORF1ab and S gene (Woo et al. 2005). At the same time, there are other subtle differences in the genome structure of CoVs in different genera. For example, alpha-CoVs and beta-CoVs usually have redundant genes between S and E, such as ORF3 and ORF4, while the genomes of delta-CoVs do not have these redundant genes between S and E, making their genomes more compact (Brian and Baric 2005; Chen et al. 2020b). In addition, the number of redundant proteins encoded by different CoVs varies greatly (Fig. 5) (Brian and Baric 2005).

## Other suborders of *Nidovirales* order

In addition to the suborder *Cornidovirineae*, the *Nidovirales* order contains 7 more suborders: *Ab-*, *Ar-*, *Mes-*, *Mo-*, *Na-*, *Ro-* and *Tor-nidovirineae* (Walker et al. 2019). Members of these suborders and their hosts will be briefly introduced here.

### Suborder *Abnidovirineae*

Currently, there is only 1 species, *Aplysia abyssovirus 1*, in the subgenus *Aplyccavirus*, genus *Alphaabyssovirus*, subfamily *Tiamatvirinae*, family *Abyssoviridae*, in the *Abnidovirineae* suborder (Fig. 1, Table 3). In 2018, by transcriptome studies, a nidovirus-like sequence assembly was detected in the marine gastropod *Aplysia californica* (California sea hare) and was named Aplysia abyssovirus (AAbV), which is the only member of the *Abnidovirineae* suborder (Bukhari et al. 2018). The genomic length of AAbV (likely the complete genome) is 35.9 kb.

### Suborder *Arnidovirineae*

**Fig. 6:**
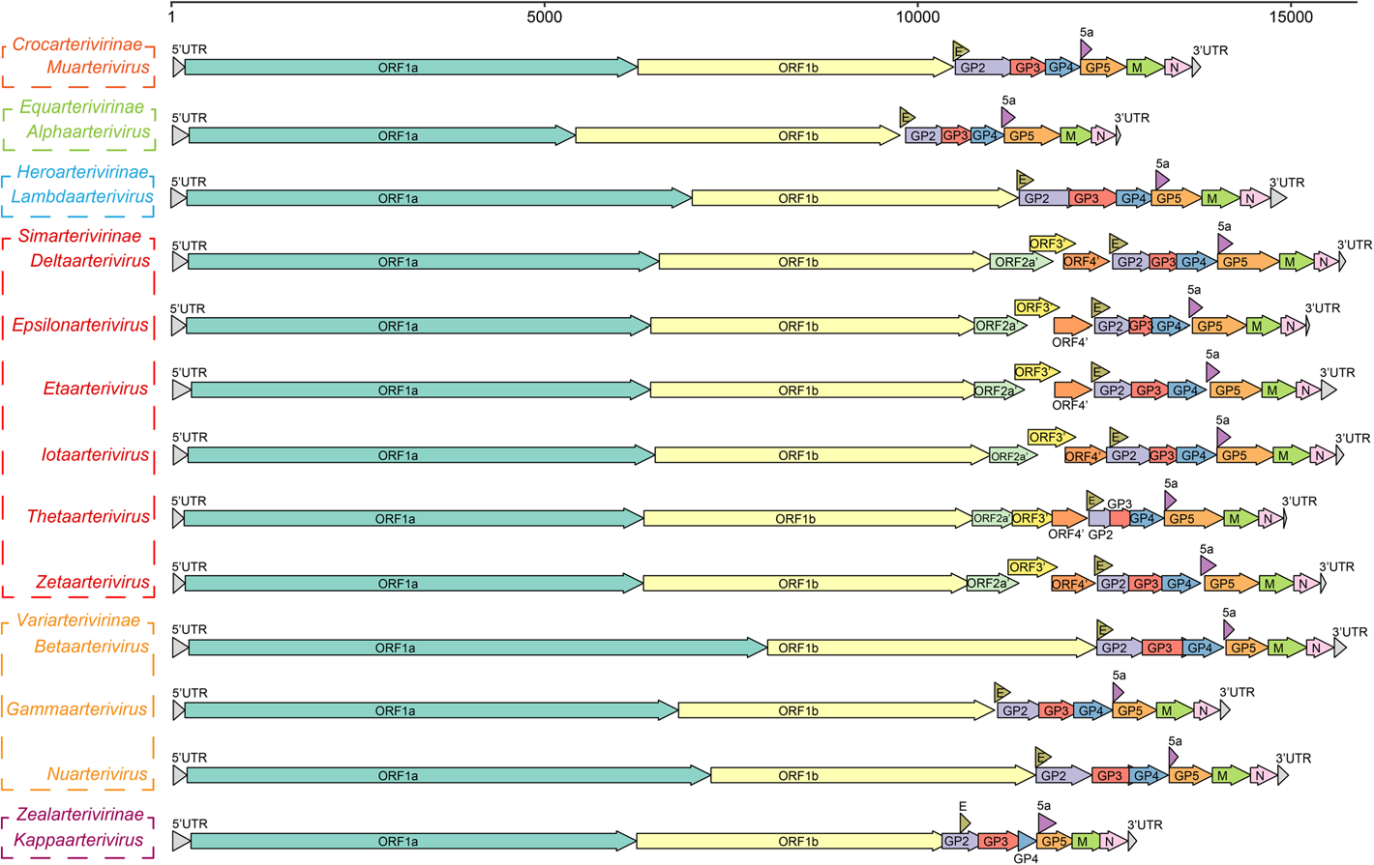
Gene composition of viruses in 13 viral genera in *Arteriviridae*. The reference strains used for mapping in each genus are as follows: *Muarterivirus* (Olivier's shrew virus 1**,** NC_035127.1), *Alphaarterivirus* (Equine arteritis virus, NC_002532.2), *Lambdaarterivirus* (Forest pouched giant rat arterivirus, NC_026439.1), *Deltaarterivirus* (Simian hemorrhagic fever virus, NC_003092.2), *Epsilonarterivirus* (Free State vervet virus, NC_029992.1), *Etaarterivirus* (Kibale red colobus virus 2, NC_034455.1), *Iotaarterivirus* (DeBrazzas monkey arterivirus, NC_026509.1), *Thetaarterivirus* (Kafue kinda chacma baboon virus, NC_029053.1), *Zetaarterivirus* (Kibale red colobus virus 1, NC_033553.1), *Betaarterivirus* (Rat arterivirus 1, NC_028963.1), *Gammaarterivirus* (Lactate dehydrogenase-elevating virus, NC_001639.1), *Nuarterivirus* (Rodent arterivirus, KY369969.1), *Kappaarterivirus* (Wobbly possum disease virus, NC_026811.2)

The suborder *Arnidovirineae* contains 4 families, *Arteriviridae*, *Gresnaviridae*, *Olifoviridae* and *Cremegaviridae*. Viruses in this suborder have the smallest genomes, about 12-16 kb, compared to viruses in other suborders (van Marle et al. 1999). The *Arteriviridae* family is divided into 6 subfamilies and contains many important pathogenic viruses (Figs. 1 and 6, and Table 2). The host range of viruses in *Artiviridae* is extensive, including mammals and reptiles (Vanmechelen et al. 2018). Most infect mammals, including horses, donkeys, rats, monkeys, baboons, pigs, etc., and others infect reptiles, such as snakes and turtles (Lyu et al. 2019). Equine arteritis virus (EAV) (Zhang et al. 2008), porcine reproductive and respiratory syndrome virus (PRRSV) (Balasuriya and Carossino 2017), and simian hemorrhagic fever virus (SHFV) (Zeng et al. 1995; Bailey et al. 2016a) cause diseases in animals and are important pathogens in the *Artiviridae* family. Recently, some novel artiviruses, such as Trionyx sinensis hemorrhagic syndrome virus (TSHSV), infecting *Pelodiscus sinensis* (Chinese soft-shell turtle), have been identified (Lyu et al. 2020). Hosts of *Gresnaviridae*, *Olifoviridae*, and *Cremegaviridae* families are all reptiles. Each family has only 1 viral species, *Ptyasnivirus 1*, *Oligodon snake nidovirus 1*, and *Chinturpovirus 1,* whose hosts are *Cyclophiops major*, *Oligodon formosanus*, and *Mauremys megalocephala*, respectively (Table 2) (Shi et al. 2018). Diverse viruses in the *Simarterivirinae* subfamily have been identified, and all of them infect nonhuman primates, such as monkeys and baboons (Fig. 6, Table 2) (Lauck et al. 2013; Bailey et al. 2014; Lauck et al. 2015; Bailey et al. 2016a). At present, no similar viruses have been detected in humans. However, humans, monkeys and baboons are primates, and it’s necessary to strengthen the monitoring of artiviruses to prevent cross-species transmission of these viruses to humans (Bailey et al. 2016b).

### Suborder *Mesnidovirineae*

The suborder *Mesnidovirineae* contains *Medioniviridae* and *Mesoniviridae* families. *Medioniviridae* consists of *Medionivinae* and *Tunicanivirinae* subfamilies with marine invertebrate hosts. *Medionivinae* has one species, *Turrinivirus 1,* detected in Turritella sea snails, and *Tunicanivirinae* has 1 species, *Botrylloides leachii nidovirus,* detected in *Botrylloides leachii* (Shi et al. 2018). Comparatively, *Mesoniviridae* contains 10 viral species, which primarily infect mosquitoes, such as Culex, Aedes and uranotaenia, but some of them have also been detected in rats (Fig. 1, Table 3) (Zirkel et al. 2011; Lauber et al. 2012; Diagne et al. 2017). Aedes and Culex mosquitoes are important hosts of arboviruses (da Silva Ferreira et al. 2020). For example, yellow fever virus and dengue virus are mainly transmitted by Aedes. Culex can transmit Japanese encephalitis virus and other viruses. More attention should be paid to some viral strains of the *Namcalivirus* subgenus in the *Alphameaonivirus* genus, such as *Hexponivirinae* subfamily, and *Mesoniviridae* family, which can be transmitted between mosquitoes and mammals (Table 3) (Vasilakis et al. 2014).

### Suborder *Monidovirineae*

The suborder *Monidovirineae* has 1 viral species, *Planidovirus 1*, in the *Dumedivirus* subgenus, *Alphamononivirus* genus, *Mononivirinae* subfamily, and *Mononiviridae* family. *Planidovirus 1* has only 1 viral strain named planarian secretory cell nidovirus (PSCNV), which was detected in the transcriptome sequences of *Schmidtea mediterranea* (freshwater planarian) (Table 3). The detected genome of PSCNV reaches 41.1 kb in length, which is the largest RNA genome characterized so far (Saberi et al. 2018).

### Suborder *Nanidovirineae*

The suborder *Nanidovirineae* is divided into 2 viral families, *Nanghoshaviridae* and *Nanhypoviridae*. To date, nanidoviruses have only been found in 2 types of fish. There is only 1 virus in each family, *Nangarvirus 1* (13.2 kb genome) in *Nanghoshaviridae* detected in *Chimaera sp.*, and *Halfbeak nidovirus 1* (18.2 kb genome) in *Nanhypoviridae* found in *Hyporhamphus sajori* (Japanese halfbeak) (Table 3) (Shi et al. 2016; Shi et al. 2018).

### Suborder *Ronidovirineae*

The suborder *Ronidovirineae* contains 2 viral families, *Euroniviridae* and *Roniviridae*. *Ronidovirineae,* named “rod-shaped nidovirus”, is 150-200 nm long and approximately 60 nm thick (Boonyaratpalin et al. 1993). There are 3 viral species in the *Tipravirus* subgenus, *Okavirus* genus, and *Okanivirinae* subfamily in the *Roniviridae* family. *Gill-associated virus* (GAV), *yellow head virus* (YHV), and *Okavirus 1* share a close genomic size of approximately 26 kb and infect different shrimp species, including *Penaeus monodon* (black tiger shrimp), *Penaeus chinensis*, and *Penaeus semisulcatus* (Cowley et al. 2004; Ma et al. 2009; Dong et al. 2017). Due to extensive aquaculture and the exchange of aquatic products, these viruses have been detected in many areas of the world with a variety of viral types, some of which are important aquatic pathogens (Soowannayan et al. 2013). There are 3 viral species in the *Euroniviridae* family, all of which have been detected in crustaceans (Shi et al. 2016).

### Suborder *Tornidovirineae*

The suborder *Tornidovirineae* has a wide range of hosts, including fish, reptiles and mammals (Table 3). The *Tornidovirineae* suborder contains 1 viral family, *Tobanivirdae,* which has been divided into 4 subfamilies (Walker et al. 2019). Among them, viruses of *the Piscanivirinae* subfamily infect fish, such as white mullet and crucian carp (Schütze et al. 2006; Batts et al. 2012), viruses of the *Remotovirinae* subfamily infect cattle (Tokarz et al. 2015), viruses of the *Serpentovirinae* subfamily infect snakes and lizards (Stenglein et al. 2014; O’Dea et al. 2016; Shi et al. 2016), and viruses of the *Torovirinae* subfamily infect diverse mammals (including porcine, sheep, goat, horses, cattle and *Tasmanian devil*) and reptiles (snakes and turtles) (Pignatelli et al. 2010; Sun et al. 2014; Shi et al. 2018; Stewart et al. 2018; Chong et al. 2019; Li et al. 2020a). The subfamily *Torovirinae* was once classified into the family *Coronaviridae*, and the prototype species of torovirus is equine torovirus (EToV), which was identified in the 1970s (Lauber et al. 2012). In the 1980s, porcine torovirus (PToV) and bovine torovirus (BToV) were detected (Pignatelli et al. 2010). Subsequently, torovirus was found in a large range of domestic ungulates, such as cattle, goats, sheep, pigs and horses, with worldwide distribution, causing acute self-limiting gastroenteritis and a significant burden to the animal industry (Hoet and Saif 2004). Currently, there are 3 species BToV, EToV and PToV in the subgenus *Renitovirus* of genus *Torovirus* in the subfamily *Torovirinae* (King et al. 2018). The recently identified toroviruses in snakes and turtles have not yet been classified (Shi et al. 2018).

## Conclusions and perspectives

In this century, knowledge about the universality, diversity, genetic evolution and host range of viruses has greatly expanded (Ge et al. 2012; Shi et al. 2016; Shi et al. 2018). Together with other knowledge regarding viruses, these findings have led to new standards and definitions of viral classification, and thus, the virus classification system recommended by ICTV is frequently being upgraded. In 2020, ICTV expanded the virus classification system from the old 5-rank hierarchy structure (from order to species) to the new 15-rank hierarchy structure (from realm to species) (International Committee on Taxonomy of Viruses Executive 2020). Viruses classified into the order *Nidovirales* synthesize 3CLpro, NiRAN, RdRp, ZBD and HEL1 proteins. Classification of nidovirus was also replaced by the new standard, and the latest classification of nidoviruses is based on sequence similarity of 5 genes, 3CLpro, NiRAN, RdRp, ZBD and HEL1 domains of the replicase protein, replacing the 7 previous domains of ADRP, 3CLpro, RdRp, NSP13, NSP14, NSP15 and NSP16. The lower ranks, including suborder, family, subfamily, genus, subgenus and species, are classified according to the overall identity of the concatenated domains of 3Clpro-NiRAN-RdRp-ZBD-HEL1. Thresholds of the demarcation criteria for each level are 73.4%, 68.3%, 51.9%, 36%, 14.7% and 7.6%, respectively (International Committee on Taxonomy of Viruses Executive 2020).

At present, the order *Nidovirales* consists of 8 suborders including 109 species, which have a wide range of hosts from invertebrates (crustaceans and arthropods) to vertebrates (fishes, reptiles, amphibians, birds and mammals) (Saberi et al. 2018). Viral genome size and structure vary greatly among different suborders in the order *Nidovirales.* For example, genome of viruses in the suborder *Arnidovirineae* is usually less than 17 kb, while that of viruses in the suborder *Monidovirineae* is 41 kb, which is the largest RNA viral genome characterized at present (van Marle et al. 1999; Saberi et al. 2018). Viruses of the subfamily *Simarterivirinae* in the suborder *Arnidovirineae* have 3 additional genes, ORF2a', ORF3' and ORF4', compared to viruses in other subfamilies in suborder *Arnidovirineae* (Lauck et al. 2013). Notably, viruses in subfamily *Simarterivirinae* infect primates, and the additional genes may function to promote viral adaption to primate hosts (Bailey et al. 2014). The abundant virus types, wide host range, consistency and variation of genome and its coding components of nidoviruses may provide information about the evolutionary path, mutation mode, host adaptation and coevolution of these viruses, which provides excellent research materials for virus tracing and other virological studies. In this review, only viruses with a clear taxonomic status proposed by ICTV were listed and introduced, while many viral strains with sequences but without a clear taxonomic status were not referenced to avoid potential ambiguity. In the future, identification and classification of additional related viruses will help to better understand the origin and evolution of nidoviruses.

CoVs can be hosted in 45 species and are a large group in the order *Nidovirales*, which contains many pathogens that infect humans, poultry and livestock, posing great threats to public health and livestock farming (Jackwood et al. 2012; Graham et al. 2013; Cui et al. 2019). Previous studies on CoVs, especially in bats, rodents, poultry and livestock, have revealed relative host specificity of CoVs. For example, SARSr-CoV primarily exists in *Rhinolophus* bats, and in particular, the most relevant bat SARSr-CoV with human SARS-CoV virus only exists in *Rhinolophus sinicus*, while the most relevant virus to SARS-CoV-2 was detected in *Rhinolophus affinis* (Ge 2013; Zhou et al. 2020b). However, CoV has its own characteristics of rapid genetic variation due to the lack of sufficient correction in genomic RNA replication and frequent genome recombination, leading to diversity among CoVs (Brian and Baric 2005). In natural reservoirs, such as bats, rodents and birds, CoVs are endemic and show high genetic diversity (Wang et al. 2015; Wu et al. 2016; Ge et al. 2017; Tsoleridis et al. 2019). However, their rapid evolution and habitat crossover among animals lead to continuous and accidental cross-species transmission (Hu et al. 2015).

Research on SARS-CoV, SARS-CoV-2, and MERS-CoV has revealed a wide range of potential hosts for these viruses (Barlan et al. 2014; Wang et al. 2020a). For instance, SARS-CoV-2 can infect and cause diseases in humans, dogs, cats, ferrets and other animals (Oude Munnink et al. 2021). It has also been reported that human contact with other animals caused by long-term viral epidemics can lead to spread of the virus among different species, and sharing habitats is crucial for breaking interspecies barriers and the subsequent interspecific transmission of viruses (Oude Munnink et al. 2021). Because of the high diversity of CoVs carried by bats, rodents, and other animals and the widespread use of their host animals, monitoring natural CoVs and keeping a distance from wild animals are critical to prevent the transmission of CoVs to human habitats (Li et al. 2020c).

The outbreak of several CoV pandemics in this century reminds us of the great threat of epidemic CoVs. The long-term prevalence of SARS-CoV-2 is partially due to the lack of attention to viral diseases and insufficient knowledge regarding pathogenic viruses. Therefore, it’s important to popularize knowledge of virology, especially about viral diseases, in the public. Moreover, new preventive techniques and therapies developed according to the characteristics of viral pathogens will greatly contribute to the control of infectious diseases.
